# Gold nanoparticles and gold nanorods in the landscape of cancer therapy

**DOI:** 10.1186/s12943-023-01798-8

**Published:** 2023-06-21

**Authors:** Prashant Kesharwani, Ruiyang Ma, Liang Sang, Mahak Fatima, Afsana Sheikh, Mohammed A. S. Abourehab, Neelima Gupta, Zhe-Sheng Chen, Yun Zhou

**Affiliations:** 1 Department of Pharmaceutics, School of Pharmaceutical Education and Research, Jamia Hamdard, New Delhi, 110062 India; 2 Department of Pharmacology, Saveetha Dental College, Saveetha Institute of Medical and Technical Sciences, Saveetha University, Chennai, India; 3 Department of Otorhinolaryngology, The First Hospital of China Medical University, Shenyang, China; 4 Department of Ultrasound, The First Hospital of China Medical University, Shenyang, China; 5 Department of Pharmaceutics, College of Pharmacy, Umm Al-Qura University, Makkah, 21955 Saudi Arabia; 6 Dr. Harisingh Gour Vishwavidyalaya (A Central University), Sagar, Madhya Pradesh 470003 India; 7 Department of Pharmaceutical Sciences, College of Pharmacy and Health Sciences, St. John’s University, New York City, NY 11439 USA; 8 Department of Ophthalmology, The First Hospital of China Medical University, Shenyang, China

**Keywords:** Gold nanoparticles, Gold nanorods, Cancer, Photothermal therapy, Chemotherapeutics, Reactive oxygen species

## Abstract

Cancer is a grievous disease whose treatment requires a more efficient, non-invasive therapy, associated with minimal side effects. Gold nanoparticles possessing greatly impressive optical properties have been a forerunner in bioengineered cancer therapy. This theranostic system has gained immense popularity and finds its application in the field of molecular detection, biological imaging, cancer cell targeting, etc. The photothermal property of nanoparticles, especially of gold nanorods, causes absorption of the light incident by the light source, and transforms it into heat, resulting in tumor cell destruction. This review describes the different optical features of gold nanoparticles and summarizes the advance research done for the application of gold nanoparticles and precisely gold nanorods for combating various cancers including breast, lung, colon, oral, prostate, and pancreatic cancer.

## Introduction

Cancer is the world’s second most prominent cause of mortality, trailing only cardiovascular disease [1–3]. Early diagnosis and treatment for cancer remain a technological roadblock at the moment. Despite major advancements in classic treatment options including radiation and chemotherapy cancer treatment remains far from ideal due to several drawbacks [2–7]. Prevailing cancer treatments frequently face challenges such as nonspecific systemic antitumor agent distribution, insufficient drug levels reaching the tumor site, unendurable cytotoxicity, restricted ability to monitor therapeutic outcomes, and the advancement of drug resistance [7–11]. Existing prognostic and diagnostic systems are inadequate to predict treatment efficacy and patient outcomes. As a result, there is an urgent need and significant opportunity to develop new and innovative technologies that could aid in delineating tumor margins, identifying residual tumor cells and micrometastases, and determining whether a tumor has spread. Nanotechnology-enabled cancer testing and therapeutics have been created to enhance specificity in the treatment of cancer [12–18]. Since clinical approval of the first micellar medication Sandimmune® and the first polymer-drug nanoconjugate Adagen® by the US FDA in 1983 and 1990, respectively, nanoparticles—materials with dimensions between 10^9^ and 10^8^ m—have been systemically administered in humans. Since then, there has been a tremendous increase in research into nanoscale therapeutic and diagnostic agents, leading to the development of a variety of biomedical nanotechnologies and platforms, including micelles, inorganic nanoparticles, dendrimers, liposomes, protein-drug nanoconjugates, and various polymer-drug nanoconjugates [19]. More than 20 bio-diagnostic or therapeutic nanotechnology-based systems have received clinical approval, and another 250 are now through clinical development. By 2015, it is anticipated that the global market share for biomedical nanotechnologies will reach US$70–160 billion, potentially competing with the existing global market for biologics. These nanoscale structures offer a variety of various, fundamentally novel features that can be used to enhance how we identify, treat, and keep track of disease conditions. Additionally, the distinctive interactions between these nanoscale materials and similarly sized physiological structures, such as proteins, organelles, and DNA, can be used to supplement current medical diagnostic and therapeutic approaches and to encourage the development of fresh, possibly more effective ones [20].

The integration of nanoparticles of noble metals and light sources has piqued the interest of researchers as a promising approach for impactful cancer treatments and diagnosis at all stages [21]. Gold/Au nanoparticles (AUNPs) have received the most attention among metallic nanoparticles (e.g., Au, Ag, Cu, and Fe16) due to their well-established biocompatibility, simple method of preparation, and stability [22–26]. Gold has high electron content and is one of the metals with the lowest chemical reactivity. Due to these advantages, AUNPS are suitable imaging agents and are often easy to locate in dynamic structures like tissue. Based on their size and morphology, AUNPS exhibit optical characteristics such as strong scattering and absorbance in the visible-near-infrared (VIS–NIR) region [13, 27–31].

Gold nanorods (GDNDs) a type of capsule-like nanoparticle, with a pseudo-one-dimensional structure and size ranging in nanometers, have become one of the thriving nanoparticles of choice of late [29]. With the advancement of synthetic methods, a wide range of materials has been attached around gold nanorods to achieve unexpected or improved plasmonic properties and to investigate cutting-edge technologies. This article highlights the latest advancements in cancer therapy, describe the benefits of gold complexes structured as nanoparticles, specifically GDNDs in different types of cancer including breast, colon, lung, prostate cancer, etc., and evaluates recent research findings in the biomedical sector.

## History of gold nanoparticles

In the fourth century A.D., gold was also used for ornamenting cups and vessels. The famous “Lycurgus Cup”, which under direct sunlight showed greenish-yellow color and when light shown throughout, the color changed to ruby red was a mystery for years [32]. Until 1857, when the scientist Michael Faraday hinted that the extremely fine particles of gold are the reason behind the cup emitting intense red color [33]. He explained the light-scattering phenomenon of suspended gold microparticles and coined the term “Faraday-Tyndall effect” for this phenomenon [34]. After almost half-century later, Hirsh and team discovered that upon irradiating AUNPs with electromagnetic waves of approximately 820 nm wavelength, the temperature of the surroundings increases. Thus they concluded that this property of AUNPs can be used to treat solid tumors [28]. In the twentieth century, scientist Mie proposed a theory describing the optical properties of metal colloidal solutions and it was revealed that the average diametric range of gold particles was very small compared to the wavelength of visible light. Then in the 1930s, after the invention of electron microscopy, the structure and composition of AUNPs is constantly studied to better understand the characterization of their size, shape, crystalline structure, etc. [35].

## Optical properties of gold nanoparticles

Despite of high electron density, gold is one of the most chemically inert metals in bulk. AUNPs demonstrate various optical parameters and applications such as in cancer imaging and diagnosis which when analysed provide scope in overcoming the pitfalls in product development. They prove to be good candidates for imaging agents and are relatively simple to locate in complex systems such as tissue [36]. The section below discussed various optical properties of gold reduced to nanosize.

### Localized surface plasmon resonance properties

The concept of shining a light on cancer cells to detect and destroy them is a constantly growing and modifying picture. To bring this concept in clinical practice, it is important to get the light delivered at the targeted position and in the required wavelength range [37]. Innovations in endoscopic techniques, solid-state lasers, and fiber optics allow for the feasible delivery and distribution of lasers to various internal body parts. Range of ~ 700–1200 nm wavelength also known as “the water window” is widely accepted as the fair window for detection and treatment of various malignancies as at this spectra region, relatively less light is absorbed by aqueous tissues [38]. Regarding this, AUNPs ought to be an ideal nanoparticle. They can be modified into different shapes which help them to absorb light in the given window [39]. AUNPs can absorb this light strongly and thus can be described by the localized surface plasmon resonance (LSPR) phenomenon [40]. This tunability observed in scattering wavelength under white light illumination permits AUNPs to function as scattering probes having different colors, varying from an orange color to ruby red. Large-size particles absorb less light and scatter more but upon reducing nanoparticles size, scattering also reduces and more light is absorbed [41].

AUNPs of dimensions ~ 10–100 nm length and 10–20 nm width act as absolute conductors. Upon illuminating these GDNDs with adequate frequencies of optical light, the electrons in the conduction band of gold get excited and cause the electrons to oscillate in a coherent and resonant manner. This causes massive light extinction, measured by the interaction of the incident radiation with the GDNDs and defined as the sum of absorption of the light and scattering [42]. This facility of GDNDs of tuning absorption and scattering in the visible as well as NIR region is advantageous compared to round-shaped AUNPs as they absorb light only in the visible range. Also, the low scattering efficiency of GDNDs is still adequate for using them in cell imaging in darker fields as plasmon scattering probes [35].

### Surface-enhanced Raman scattering (SERS) properties

Apart from scattering light elastically, AUNPs can augment the inelastic light scattering of the photons present in molecules. These inelastically scattered photons attain greater energy levels both rotational and vibrational and this phenomenon is known as Surface-enhanced Raman scattering (SERS). Upon getting adsorbed on a metallic nanoparticle system, the Raman signals of the molecule amplified up to several degrees of magnitude [43]. Explicitly this magnitude can be increased in the hotspots, by up to 10^14^ folds, making the detection of even a single molecule possible. Raman scattering enhancement can be explained via two mechanisms, chemical enhancement (CE) and electromagnetic enhancement (EE). Chiefly, EE has an important role here. The local enhancement of the electromagnetic field observed at the high curvature ends of AUNPs is endorsed mainly by the LSPR, resulting from the electron’s excitation in the conduction band of metallic nanoparticles [44]. To optimize the EE surface effect, the wavelength (λ) of the incident laser should be equal to the wavelength of the metallic nanoparticle’s LSPR peak.

The geometry of the nanoparticle has been extensively studied for SERS efficiency, and it has been discovered that anisotropic structures such as rods provide more SERS signals for various analytes than formally spherical nanoparticles [45]. Due to their exclusive LSPR properties, GDNDs are considered efficient substrates for SERS [46]. In a study, the SERS behavior of different shape GDNDs diluted with dispersions of Mercaptobenzoic acid (MCAD) with a varying Aspect ratio (AR) was studied. Observations were made that when the λ of the excitation source reaches the longitudinal LSPR peak λ of the GDNDs, the strongest SERS signals were exhibited [47]. In another research, 4-amino thiophenol embedded different shaped GDNDs (rods, dog-bones, cubes, spheres, tetrapod shaped) were inspected for their SERS response and the enhancement factor was reported on a scale of 103–105. Results showed that GDNDs having AR of 3.3 and the dog-bones containing 110 facets exhibit the highest enhancement factor whereas the enhancement factor of the sphere was the lowest. Also, the GDNDs with AR 3.3 has 8 folds greater enhancement factor than the 2.4 AR GDNDs because of the lighting rod impact [48]. Thus the surface of GDNDs can be easily modified and used as a recognition tool for toxin-like small-size molecules and can be used as a theranostic agent for biological systems [49].

#### Photothermal properties

The photothermal conversion ability of gold is a significant feature that helps in its biomedicine application. This conversation depends strongly upon the concentration, geometries, longitudinal plasmon λ and the state of assembly of GDNDs. GDNDs due to this property acts as an outstanding applicant for controlled chemotherapeutic release and cancer-killing agent [50]. The gold complexes using a phonon-incorporated process convert the absorbed light into heat. Precisely, once the energy from the incident electromagnetic wave gets absorbed, it conveys in the form of oscillation to the free electrons of gold. Further then it delivers to the phonon bath [51]. This complete process of energy transfer happens in picoseconds, it takes 100 picoseconds for the produced heat to transmit to the surrounding medium. A group of researchers observed that the longitudinal plasma resonance λ upon reaching close to the incident laser λ results in maximum photothermal efficiency of GDNDs [52]. The dimensions of GDNDs have an impact on the photothermal efficiency, as upon amplifying particle volume, the conversion efficiency reduces. This is because of the increasing scattering ability of GDNDs [53]. Another study represents the effects of irradiation power and GDNDs concentration on the temperature distribution during photothermal therapy (PLT). It was concluded from the study that instead of irradiation power, GDNDs concentration controlled the increase in temperature in the target tissues. Furthermore, due to thermoelastic expansion and rapid heating, gold particles upon absorbing a stronger pulsed laser led to ultrasound generation, and this is known as the photoacoustic effect (PTCI). The signals produced by this effect are called photoacoustic signal that can be converted to photoacoustic images. Thus when AUNPs gather at the site of targeting, yields a specific and sensitive pathology diagnostic image [54].

## Synthesis techniques of AUNPs

Preparing size and shape-controlled AUNPs is important for utilizing them in biological and medical activities. For synthesizing AUNPs, bottom-up and top-down are the two major opted techniques. In the bottom-up technique, nano-sized particles are formed by arranging atoms of gold in the desired dimensions and fashion. For this, the Green, Brust and Turkevich methods are used. In contrast to this, the top-down approach generates AUNPs from bulk gold that is broken down systematically to produce nanoparticles. Here, the formation of such nanoparticles is regulated by a gold matrix. Here methods such as lithography, thermal decomposition, laser ablation, sputtering, and bulk mechanical milling/ metal grinding are employed [55].

Preparing AUNPs involves 2 major steps: i) Employment of citrate-like reducing agents for reduction of gold precursor (generally aqueous gold salt solution is used) to gold nanoparticles. ii) Prepared nanoparticles are then stabilized using capping agents that hamper the agglomeration of metallic nanoparticles [39].

### Top–down approach

The top-down method is a subtractive process, beginning with the carving of bulk materials and ending with self-assembled nanoscale particles. Apart from photolithography and micropatterning, various physical methods for slicing bulk metal include radiation, thermolysis and pyrolysis. Pyrolysis is 4 steps noble metal nanoparticle-producing process that includes the production of drops from the parent solution, then the size of these drops is reduced via evaporation, and from these drops, oxides are produced to ultimately generate metal nanoparticles. However, the issue with the top-down approach is that it is difficult to control the shape and size of produced nano-sized particles during such processes, which in turn affect the physical and chemical characters of metal nanoparticles [56]. Even with the generally employed pyrolysis process, drawbacks such as low-purity products, porous film formation over nanoparticles, and limited products are associated. To overcome such conditions and to produce size-controlled particles, maintenance of high temperature and pressure throughout the process is required, thus making the whole process uneconomical and non-feasible [57].

### Bottom-up approach

Being another method of preparation of nanoparticles, the bottom-up approach depends upon coordination units of molecular structures. The bottom-up technique has been discussed below.

#### Chemical synthesis

Chemical reduction of metal ions is the most common and feasible approach of producing metal nanoparticles including AUNPs. A standard procedure of AUNPs synthesis involves reducing Au(III) ions to Au(0) atoms, that further form clusters and later, in the presence of reducing or stabilizing agent aggregate into polycrystalline large-size particles [56].

#### Turkevich method

In 1951, Turkevich and team chemically synthesized AUNPs for the first time by reducing Au (III) of HAuCl4 to Au(0) in the presence of sodium citrate. Since then, it is a widely employed process for AUNPs production. Apart from sodium citrate, reducing agents such as UV light ascorbic acid, and amino acids, also serves the purpose, and various stabilizing agents are also engaged in the process. This method produces nanoparticles of very small size (1–2 nm) and because of this limited size range, the application of the Turkevich Method was finite. Due to this, several advancements were made in the basic method by different groups of scientists to extend the size range of produced particles [39].

In 1973, scientist Frens tried modifying the Turkevich method by varying the ratio of HAuCl4 and sodium citrate to produce nanoparticles of the size range 5 to 150 nm. However, this produces particles of poor diametric range (< 30 nm) [58]. Later cm. Natan and Brown in 1998 tried using a mild reducing agent hydroxylamine to synthesize AUNPs of diameter up to 100 nm While brown in 1999 proposes the preparation of highly size and shape-controlled AUNPs by employing boiled sodium citrate. This method produces monodispersed particles of good nanometric range i.e. 20-100 nm [59].

#### The Brust method

This is a two- phase reaction method used to develop AUNPs of 1.5–5.2 nm size range by using organic solvents. The method includes the conversion of the gold salt-containing aqueous solution to organic solvent using phase-transferring agents such as tetraoctylammonium bromide. After this, gold is reduced using sodium borohydride as a reducing agent. Lastly, the obtained AUNPs are stabilized by the action of alkanethiol. This method produces air and heat-stable AUNPs having controlled size and less dispersity [60].

#### Seed-mediated growth

Both, Turkevich method and Brust method produce round-shaped AUNPs. However, seed-mediated growth allows producing rod-shaped AUNPs. For this method, firstly seeds are synthesized using salts of gold, reduced by NaBH_4_-like reducing agents. Then the seed particles are transferred to a solution of gold salt and ascorbic acid like a weak reducing agent that obstructs the nucleation and speeds up the synthesis of rod-shaped AUNPs [61].

## Characterization of AUNPs using different techniques

Core size and attached ligands are the two chief characters of AUNPs ligands that influence their hampers activity. Small AUNPs called monolayer-protected gold clusters (MP-GC) have distinct dimensions and structures and quantum properties such as quantized charging, optical absorbance bands and tunable band gaps. Thiolate-stabilized AUNPs possess a core diameter < 10 nm. Whereas, uncapped AUNPs are in the diameter range of 2–150 nm. On the other hand, large-size AUNPs show bulk gold-like properties such as “Surface Plasmon Band” (SPB) with a wavelength of 520 nm. Thus, characterizing the core size and capping agents are beneficial for analyzing the AUNPs application. For calculating size depending upon the presence or absence of SPB, UV–Vis spectroscopy is used. Thermal Gravimetric Analysis detects the amount of gold and ligand present in nanoparticles while Transmission electron microscopy presents the images of gold core. The characterization of protecting ligands can be done by Nuclear Magnetic Resonance or Fourier transform infrared spectroscopy [62]. Apart from this, to perform all the tasks altogether such as calculating molecular formula, ligands characterization, and core size measurement, Mass spectrometry is employed. The mass spectra give the number of gold atoms used in the particle formation. Also depending on the masses, it becomes affordable to find variations in the stoichiometry of different ligands [63].

## Effect of different parameters on appearance and functionality of Gold Nanoparticles

The environment, size, and physical characteristics of gold nanoparticles have a significant impact on how they interact with light. The free electrons interact with the oscillating electric fields of a light ray traveling near a colloidal nanoparticle, generating a coordinated oscillation of electron charge that is in resonance with the frequency of visible light [64]. The surface plasmon resonance phenomenon results in the absorption of light in the blue-green region of the spectrum (about 450 nm) for small (about 30 nm) monodisperse gold nanoparticles, while red light (about 700 nm) is reflected, producing a rich red colour. The wavelength of surface plasmon resonance-related absorption moves to longer, redder wavelengths as particle size rises. This results in solutions that have a light blue or purple colour because red light is absorbed and blue light is reflected [65]. The majority of visible wavelengths are reflected, giving the nanoparticles their transparent or translucent colour, while surface plasmon resonance wavelengths shift into the infrared region of the spectrum as particle size increases toward the bulk limit. The size or form of the nanoparticles can be changed to modify the surface plasmon resonance, creating particles with customized optical properties for various purposes [66].

The in vitro response of U87 glioma cells to several siRNA-conjugated gold nanoconstruct formulations of different sizes and shapes (13-nm spheres, 50-nm spheres, and 40-nm stars) that target the expression of isocitrate dehydrogenase 1 (IDH1) was compared by Yue et al. When compared to 13-nm spheres, the uptake efficiency of 50-nm spheres and 40-nm stars was significantly higher [67]. Confocal fluorescence microscopy revealed that all three formulations were localized in endosomes at the beginning of the incubation period (2 h), but that after 24 h, the 50-nm spheres and 40-nm stars were neither in endosomes nor lysosomes, while the 13-nm spheres remained in endosomes. The 13-nm spheres were confined and scattered within endocytic vesicles, whereas the 50-nm spheres and 40-nm stars were aggregated, some of which were found outside of endocytic vesicles. When comparing nanoconstructs of various sizes and shapes, larger particles (50-nm spheres and 40-nm stars) had more potential as carriers for the transport of siRNA while maintaining constant siRNA surface density and nanoparticle concentration [67].

Similarly, with an emphasis on the size-dependent effects, Xia and team examine the uptake, cytotoxicity, biodistribution, and in vivo toxicity of commercially available and well-characterized AUNPs (5 nm, 20 nm, and 50 nm) as well as their putative toxicity mechanisms in cancer cells and normal cells. Data showed that the absorption of AUNPs increased with particle size (5–50 nm) in HepG2 cancer cells but reduced in L02 normal cells. Small (5 nm) AUNPs were more cytotoxic to normal and cancer cells than larger (20 and 50 nm) ones. Intriguingly, 5 nm AUNPs primarily produced necrosis in L02 cells through the upregulation of TLR2 and the release of IL-6 and IL-1a cytokines, whereas it induced both apoptosis and necrosis in HepG2 cells through the formation of reactive oxygen species (ROS) and the activation of pro-caspase3. The treatment of 5 nm AuNPs but not 20 nm and 50 nm AuNPs resulted in an increase of neutrophils and minor hepatotoxicity in mice. Of all, 50 nm AuNPs demonstrated the longest blood circulation and maximum distribution in the liver and spleen [68].

In another study, star, rod, and triangle-shaped methylpolyethylene glycol-coated anisotropic gold nanoparticles were developed. Analyzing how these nanoparticles were internalized by RAW264.7 cells allowed for a parametric assessment of the influence of shape. From least to greatest, stars, rods, and triangles were shown to have the most effective cellular uptake of the gold nanoparticles. Examining the three types of gold nanoparticles’ potential uptake methods, it was discovered that different shapes tended to adopt distinct endocytosis pathways in varying amounts [69].

## Clinical pathway of AUNPs and GDNDs

The clinical translation of AUNPs and GDNDs is still under exploration. The first clinical trial on AUNPs was conducted in the year 2006 and was named CYT-6091. It is tumor necrosis factor-α conjugated and polyethylene glycol (PEG) decorated AUNP, that entered into early-phase (NCT00436410) clinical trials and phase I (NCT00356980, NCT00436410) [70, 71] clinical trial. The pre-clinical study demonstrated a reduction in SCC VII head and neck tumor squamous cell carcinoma and 4T1 breast cancer cells using a combined approach of AUNP and radiation therapy. The phase I clinical trial conducted on various cancer patients including breast, colon, and pancreatic ductal cell adenocarcinoma demonstrated no dose-limiting toxicity.

Considering the diagnostic ability of AUNPs, another clinical trial (NCT04907422) was conducted to provide highly sensitive biomarkers for the early detection and treatment of salivary gland cancer to improve the patient’s prognosis and outcome. The CD24 primer was conjugated with AUNP and showed the effectiveness of therapy in assessing the prognosis of salivary gland tumors [72]. Intending to achieve improved survival rates and reduced healthcare expenditures/patient, the forthcoming of cancer prevention depends on the prompt identification and surveillance of precancerous lesions. Amal et al. developed non-invasive diagnostic sensor which can distinguish the lung cancer patient from healthy individuals. The study was conducted in four phases on 40 lung cancer patients and 56 healthy volunteers. In the initial phase of the study, the ‘offline method’ was used to collect the exhaled alveolar breath from healthy volunteers and lung cancer patients. The method was designed in a way to evade the barriers associated with distinguishing endogenous components from exogenous components in the breath. Endogenous volatile organic components (VOC) are the product of biochemical cycles of the cell that provide an insight into the biological system while exogenous VOC can either directly absorbed from the lung or through the skin or blood. In the second phase, the gas chromatography-mass spectrometry (GC–MS) technique was used to detect relative components of VOCs, which served as biomarkers for lung carcinoma. In the next phase, 9 cross-reactive chemiresistors were developed wherein each sensor was accountable for numerous odorants (obtained via breath testing) for the detection of lung cancer. The chemiresistors were functionalized on 5 nm gold nanoparticles having variable organic moieties such as decanethiol, 4-methoxy-toluenethiol, 11-mercapto-1-undecanol, dodecanethiol, 1-butanethiol, 2-mercaptobenzoxazole and tert-dodecanethiol. Such a strategy combines the applicability of organic functionalities with the processability and robustness of inorganic agents. It was observed that sensors rapidly and reciprocally responded to various representative biomarkers of lung neoplasm at different concentrations. Sensor-based approach in a clinical study can precisely investigate biomarkers responsible for the growth of lesions, the relation between individual biomarkers with the sensitivity and specificity of sensors in a mixture of different components, and can optimize sensors without the hindrance of patient’s metabolic state, diet, or genetics. In the fourth phase, simulated patterns of breath were obtained using GC–MS technique. The results obtained showed perfect overlaps in between the actual and simulated breath samples, indicating the robustness of the simulation approach. It was also observed that water molecules hardly affected the sensitivity of AUNPs [73]. As per our research, no clinical trials were conducted on GDND as it is still a newborn in a clinical setting. Another clinical trial was conducted for targeting Bcl2L12 overexpressed glioblastoma cells as such cells are highly resistant to apoptosis. Spherical nucleic acid was decorated on AUNP to evaluate the safety profile of the drug (NU-0129), which can cross the blood–brain barrier (BBB). 0.04 mg/kg of the drug was administered intravenously in patients and was found that none of the patient showed serious adverse drug reactions (NCT03020017). AuroLase® particles were utilized in a localized therapy in a recent clinical trial to treat primary or metastatic lung cancers. (NCT01679470). In our view, the clinical data is still inadequate to be applied for marketing. The reason could be the back of sophisticated tools for manufacturing and assessment on a large scale.

## Gold nanoparticles for combating cancer

Cancer cells are highly involved in mortality worldwide. The widespread scope of nanoparticles demonstrated a ground-breaking approach for the delivery of chemotherapeutic, chemo-preventive and theranostic agents. Research being focused on novel treatment modalities encouraged us to envisage the effect of AUNPs in the treatment of various cancers. The section below demonstrates the efficacy of AUNPs in various malignancies (Table 1).

**Table 1:** Application of Gold nanorods as delivery agents in anticancer therapy

Therapeutic agent/Bio-molecule	Coupling molecule	Type of study	Type of cancer	Name of cell line	Animal model	Outcomes	Ref
Anacardiaceae	-	In vitro In vivo	Breast cancer	MCF-7 breast tumor cell line	Female BALB/c nude mice	The encapsulation of drug lead to improved bioavailability of drug as it helped the drug molecules to reach the targeted site. This led to superior cytotoxic action of AC-AUNPs further attributed to the anomalies occurring at metabolic stage and due to the prominent rate of proliferation	[74]
Indocyanine green and paclitaxel	Hyaluronic acid and cationic bovine serum albumin	In vitro In vivo	Breast cancer	4T1 tumor cells	BALB/c mice	The prepared nanoparticles act as a dual targeting agent by inhibiting tumor as well as lung metastasis	[75]
Technetium-99 m labeled-resveratrol	-	In vitro In vivo	Colon cancer	HT29 colon cancer cells	Male Sprague Dawley rats	The study establishes a base for using AUNPs to deliver potent radiolabelled drugs, limited by poor bioavailability to the targeted cancer sites as theranostic agents	[76]
SN38	Hyaluronic acid (HA)	In vitro	Colon cancer	HCT-29, SW480 and CHO cell lines	-	SN38 and HA conjugated AUNPs has a dual anti-tumor effect. The nanoparticles delivered the drug successfully at the site of action. Also the cytotoxic effect of AUNPs increases upon applying an external source of light (LED), that promotes photothermal activity of the gold core	[77]
Bovine pancreatic ribonuclease (RNase A)	-	In vitro In vivo	Colon cancer	SW-480 colon cancer cells	-	RNase conjugated AUNPs suppressed cancer cell invasion by inhibiting extracellular signal regulated protein kinases (ERK1/2) pathway	[78]
Docetaxel	folic acid	In vitro	Lung cancer	H520 lung cancer cell line	-	To increase the bioavailability of poorly soluble docetaxel, it was encapsulated in gold nanoparticles and folic acid conjugation further improve the tumor targeting ability of the nanoparticle	
EGFR siRNA	Collagen	In vitro In vivo	Lung cancer	A549 lung cancer cell line	Male nude Balb/c mice	Collagen fabrication led to enhanced siRNA loading capacity	[79]
Apoptosis protein-2 inhibitor (API-2) siRNA	Hyaluronic acid (HA)	In vitro	Lung cancer	A549 lung cancer cell line	-	API-2 siRNAs containing AUNPs -HA were taken up efficiently by A549 cells by abolish API-2 expression and other oncogenic activities, implying that API-2 siRNA or other siRNA containing AUNPs -HA nanocomposite could be used to treat cancer and other gene disorders	[80]
Specificity Protein 1-siRNA (SP1-siRNA)	-	In vitro In vivo	Lung cancer	A549 lung cancer cells	Balb/c mice,	Radiosensitizing ability of siRNA-loaded AUNPs was investigated on lung tumors, both in vitro and in vivo	[81]
Paclitaxel (PTX)	Polyethylenimine-Pluronic	In vitro In vivo	Prostate cancer	PC-3 prostate cancer cell lines	PC3 tumor-bearing mice	An effective and non-invasive therapy for androgen-resistant prostate cancer was developed using Polyethylenimine-Pluronic micelles encapsulating PTX containing AUNPs	[82]
-	Double stranded-DNA	In vitro In vivo	Prostate cancer	PC-3 and DU-145 prostate tumor cells	Balb/c mice	Activity of double stranded-DNA complexed AUNPs as vectors for reprogramming cancer cell metabolism was investigated and reported	[83]
-	Prostate-specific membrane antigen (PSMA)	In vitro	Prostate cancer	PC3pip and PC3flu cells	Balb/c mice	A multimodal contrast agent utilizing NIRF/CT/MR techniques for early-stage diagnosis and clinical application in prostate cancer exhibit excellent results in terms of PSMA targeting, effective mononuclear phagocyte system escaping, and profitable renal-clearable behaviour in animal model	[84]

### Drug delivery

Cancer cells are highly involved in mortality worldwide. The widespread scope of nanoparticles demonstrated a ground-breaking approach for the delivery of chemotherapeutic, chemo-preventive and theranostic agents. The research focused on novel treatment modalities encouraged us to envisage the effect of AUNPs in the treatment of various cancers. The section below demonstrates the efficacy of AUNPs in various malignancies (Table 1).

#### Breast cancer

A highly prevalent cancer among females is breast cancer and a prime cause of cancer-related morbidity among them. Originating from the mammary tissues, the tumor metastasizes further to the lobules or ducts of the milk-secreting gland. If detected early, it can be treated via chemotherapy, radiotherapy, etc., but if left treated, it can spread to other organs like the brain, bones, etc. [5, 9, 25, 85–90].

Green syntheses use components derived from natural sources such as, bacteria, plants, fungi, and algae, or to produce AUNPs, making the procedure less expensive and more environmentally friendly [91]. This led to a paradigm shift in AUNPs production. Pechyen et al., prepared AUNPs of Anacardiaceae peel extract using an uncomplicated photosynthesis procedure [74]. Anacardiaceae is a common tropical tree found in tropical, subtropical, and temperate climate zones. It is a frequently utilized plant that has also been used in traditional medicine systems [92]. There are certain components found in the pulp of these fruits that exhibit potential thrombolytic, cytotoxic and antioxidant activity. The peel extract contains phenols and flavonoids [93]. Various studies indicate the reducing action of these bioactive compounds and their involvement in the of AUNPs synthesis [94]. The formation of Anacardiaceae loaded AUNPs (AC-AUNPs) was implied by the visible color change during the reaction, from yellow to purple and the same was verified by UV–Vis spectroscopy. To assess the cytotoxic effect of AC-AUNPs, MCF-7 breast cancer cell line and Vero cells mimicking normal cells were used. Various concentrations of AC-AUNPs were subjected to the assay. Results indicated the better cytotoxic action of prepared AC-AUNPs as compared to control and plain drug-loaded solution and in a dose- time dependant manner and this activity was highly prominent after 72 h of treatment [74]. This superior cytotoxic action of AC-AUNPs is attributed to the anomalies occurring at the metabolic stage and due to the prominent rate of proliferation that leads to enhanced cellular uptake of the nanoparticles [95]. Post 72 h of treatment, DCFH-DA staining for the determination of ROS levels of cells was also done. The same results were obtained in this examination as ROS levels of AC-AUNPs treated cells increased in a dose-time dependant manner. This implies oxidative cell damage via ROS production. The outcomes of this experiment were in line with the past experimental results and thus emphasis on the benefits of green synthesis AUNPs encouraged DNA oxidative destruction, indicating the AUNPs mediated genotoxicity, resulting from the ROS-induced cytotoxicity [74].

**Fig. 1:**
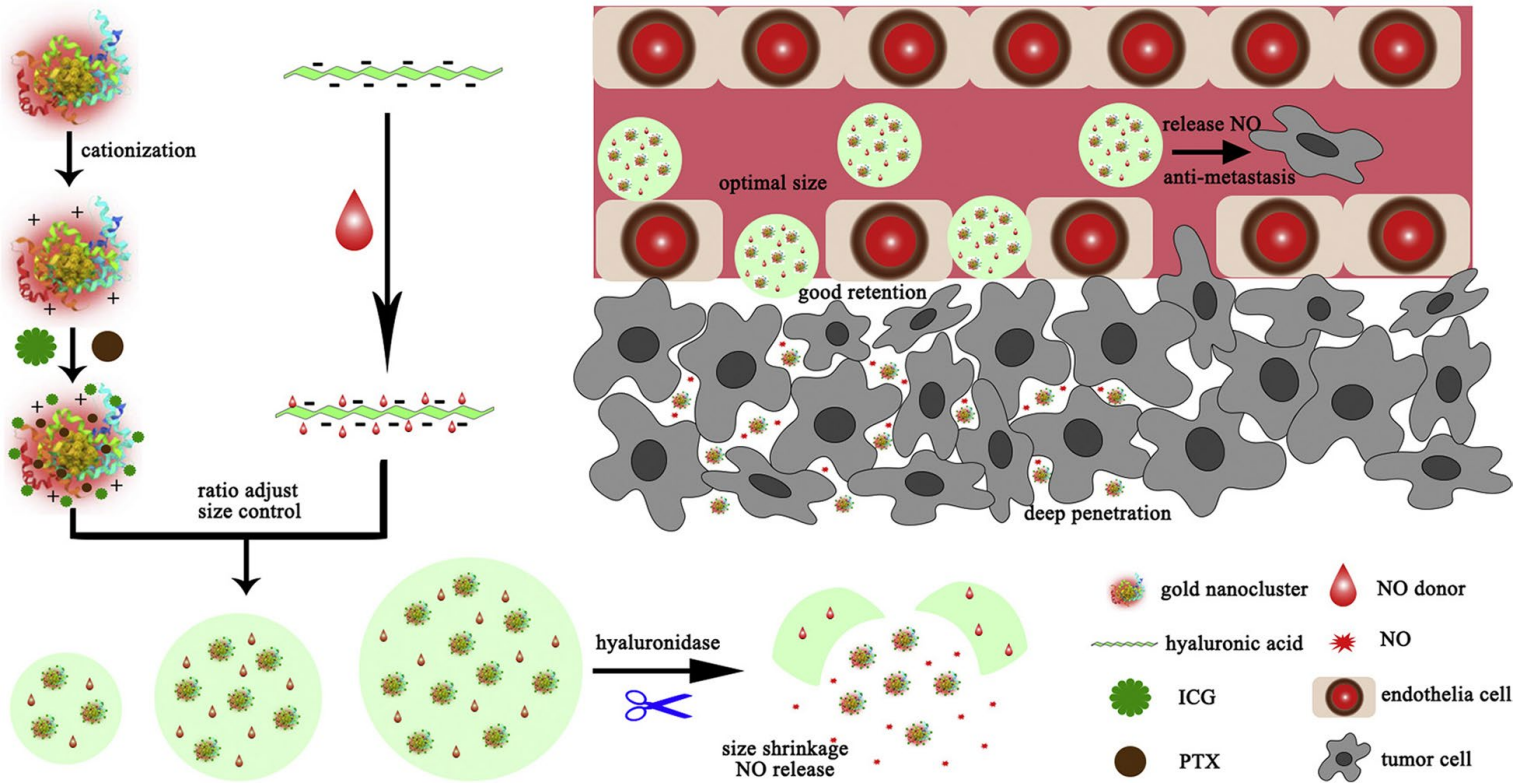
Illustration of action of size- reducible CD44 targeting, hyaluronic acid protected- red emission, renal-clearable, indocyanine green and paclitaxel loaded, cationic bovine serum albumin coated gold nanoparticles (NO_3_-ICG-PTX@ CBSA-AUNPs /HA) on tumor cells. Reproduced with permission from reference [75]

**Fig. 2:**
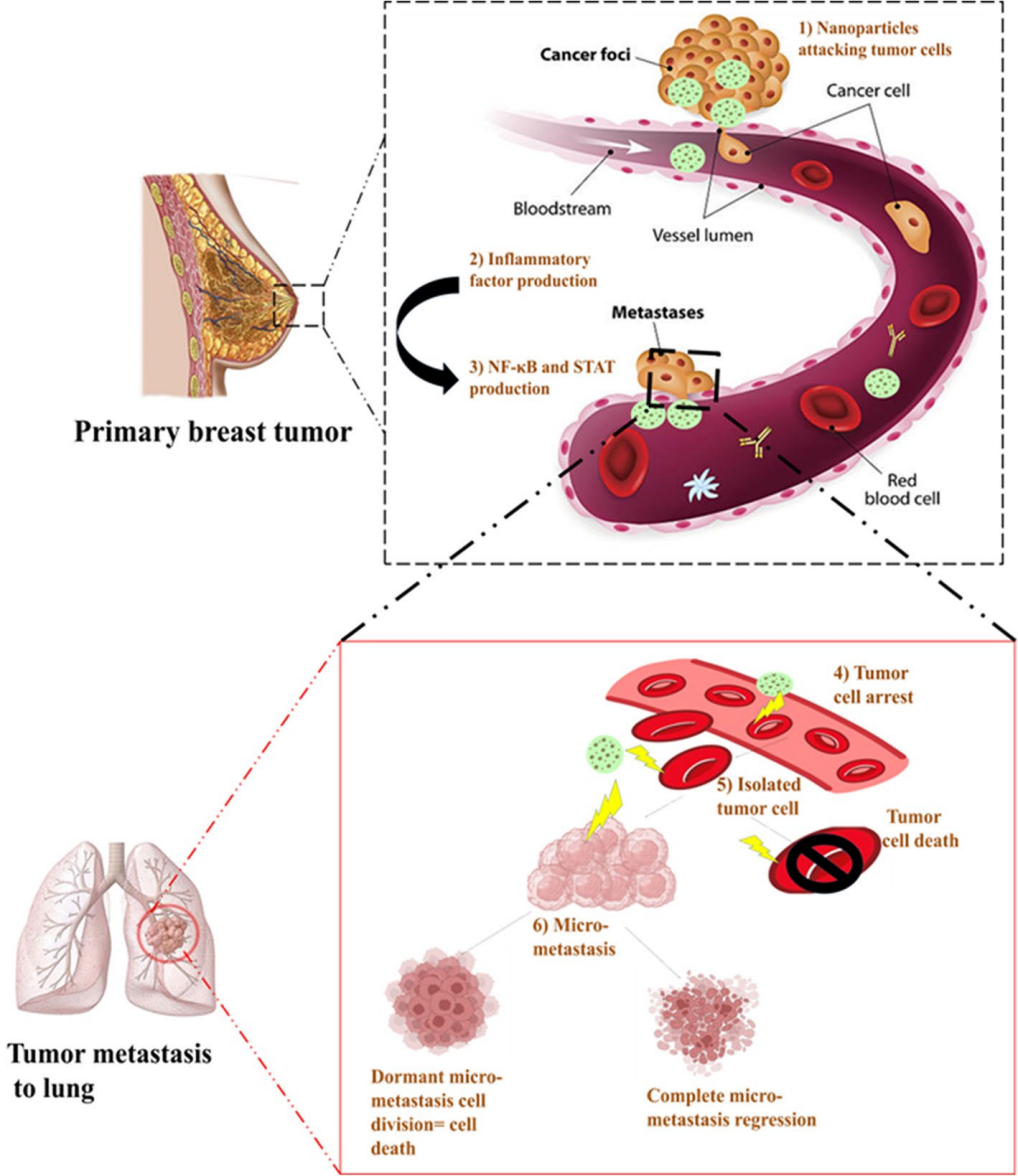
Illustrating the primary and metastasis cancer and action of CD44 targeting, hyaluronic acid protected- red emission, renal-clearable, indocyanine green and paclitaxel loaded, cationic bovine serum albumin coated gold nanoparticles on primary breast tumor as well as metastatic lung tumor cells

Dimensional properties are an important aspect of nanoparticles for employing the enhanced permeability and retention (EPR) effect in tumor targeting. Particles of small size possess good penetration but poor retention while large-size particles are contrary to this. For overcoming this issue, nanoparticles with size- reducible property is being designed however, their size at the initial stage along with the complex microenvironment of the tumor cells; hamper the nanoparticle’s distribution in the tumor [96]. In respect to this, a team of researchers utilized small-sized CD44 targeting, hyaluronic acid protected- red emission, renal-clearable, cationic bovine serum albumin coated gold nanoparticles (CBSA-AUNPs /HA). By varying proportions of hyaluronic acid and CBSA-AUNPs, CBSA-AUNPs /HA nanoparticles of different sizes were formulated. After optimization, CBSA-AUNPs /HA of 200 nm showing optimum EPR effect was selected to incorporate indocyanine green and paclitaxel for photothermal- chemo therapy and nitric oxide (NO), regulating TME thus augmenting drug delivery (NO_3_-ICG-PTX@ CBSA-AUNPs /HA) (Fig. 1). Then their pharmacokinetic profiles and tumor-targeting efficiencies were assessed. The complex upon encountering hyaluronidase, exhibit size-reducible property, showed intra-tumor accumulation, gets evenly distributed in breast cancer cells, suppress tumor growth by up to 95% and prevented lung metastasis by 88% (Fig. 2). Overall, the strategy provides a dual effect as an anti-tumor and anti-metastatic agent [75].

**Fig. 3:**
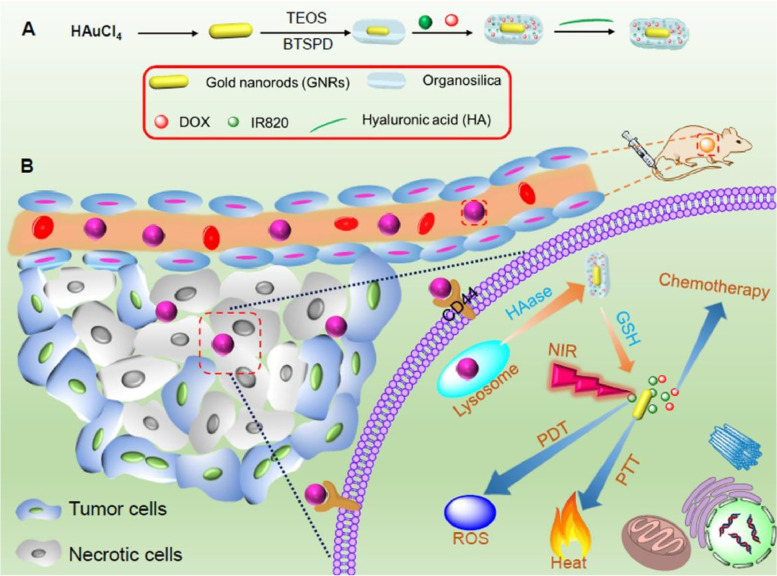
Schematic representation of (**A**) IR820/DXB-MO@HA preparation (**B**) IR820/DXB-MO@HA targeting mammary cancer producing Redox/Enzme-Responsive drug release. Reproduced with permission from reference [97]

Cheng and co-workers formulated a NIR laser-aided dual-responsive hybrid GDNDs for the treatment of breast cancer. They co-loaded these hybrid GDNDs with chemotherapeutic agent doxorubicin (DXB) and photosensitizer (IR820) and coated them externally with mesoporous organosilica, a bio-degradable material. Furthermore, the whole assembly was encapsulated within hyaluronic acid (HA) to enhance the biocompatibility and targeting ability of the prepared GDNDs (IR820/DXB-MO@HA) and also to avoid the premature drug release from the nano-hybrid system during the systemic circulation [97]. HA is supposed to interact with CD44 + receptors, highly expressed on the cell-membrane of various cancer cells [98] and this interaction will lead to cellular uptake of hybrid GDNDs specifically inside the tumor cells (Fig. 3).

Upon laser irradiation of 808 nm, NIR longitudinal surface plasmon resonance (LSPR) of photosensitizer IR820 and GDNDs [99] simultaneously produce both singlet oxygen and heat respectively. Hence, the hybrid nano-system could produce triple-modal-chemotherapy/Photo-dynamic/Photo-thermal combined cancer treatment [97]. After accumulation inside tumor cells, organosilica and HA are expected to get degraded by the action of glutathione (GSH), and hyaluronidase (HAase) respectively, causing the dual (redox and enzymatic) triggered intracellular release of IR820and DXB [100]. To confirm the same, a cellular uptake study was performed and the distribution of IR820 and DXB was checked using 4T1 Murine mammary adenocarcinoma cells [97]. DXB due to its small molecular size, upon excitation at 485 nm emits bright fluorescence of red color [101]. CLSM images of 4T1 cells treated with IR820/DXB-MO@HA showed the quenching of the red fluorescence of DXB due to the surface plasma resonance of GDNDs [102]. However, after treating these cells with GSH, the red fluorescence of DXB was visible. This is because the degradable silica contains disulfide bonds which were broken by the action of GSH, and therefore the chemotherapeutic drug got released and marked its red fluorescence. This predicted that the loaded chemotherapeutic agents would be released from the hybrid nano-system, once inside cancer cells [97]. Moreover, the anti-tumor efficacy of the prepared system was assessed in vivo 4T1 tumor-bearing female BALB/c nude mice xenograft model.

Animals treated with IR820/DXB-MO@HA + NIR laser irradiation exhibit significantly greater tumor growth retardation with three out of five mice showing complete resolution of tumor burden, compared to the animals administered with IR820/DXB-MO@HA alone. Later, the organs of the treated animals were subjected to H&E staining to assess the uptake of the drug by other organ tissues and also to assess the cardio-toxicity caused by DXB. No damage to cardiac tissues was reported signifying the biosafety and biocompatibility of the prepared nano-system. Altogether, the results revealed excellent therapeutic efficacy of tri-modal combined IR820/DXB-MO@HA with NIR laser irradiation [97].

Hypoxia, a condition of constrained oxygen supply is a major biological obstacle in cancer treatment [103]. Many solid tumors comprise hypoxic cells that show resistance against various chemotherapeutic drugs. This happens as certain genes engaged in drug resistance are up-regulated while DNA repair pathways get down-regulated, also the sensitivity towards p53-mediated apoptosis is diminished in such cells [104]. The anti-tumor activity of chemotherapeutics and photosensitizers that works by converting oxygen present in the tumor cells to singlet oxygen or Reactive oxygen species (ROS) which further leads to the demolition of other cellular components of cancer cells, including DNA is certainly impaired in hypoxic conditions [105, 106].

Photosynthesis is a phenomenon that utilizes light energy and converts water and carbon dioxide into sugars, producing oxygen as a by-product. Single-celled, spherical-shaped eukaryotic microalgae grow in fresh water and contain chlorophyll, a photosynthetic pigment that helps in conducting effective photosynthesis. Chlorella is one such genus of microalgae and can generate oxygen effectively once provided with an adequate quantity of carbon dioxide, water, minerals, and sunlight [107].

**Fig. 4:**
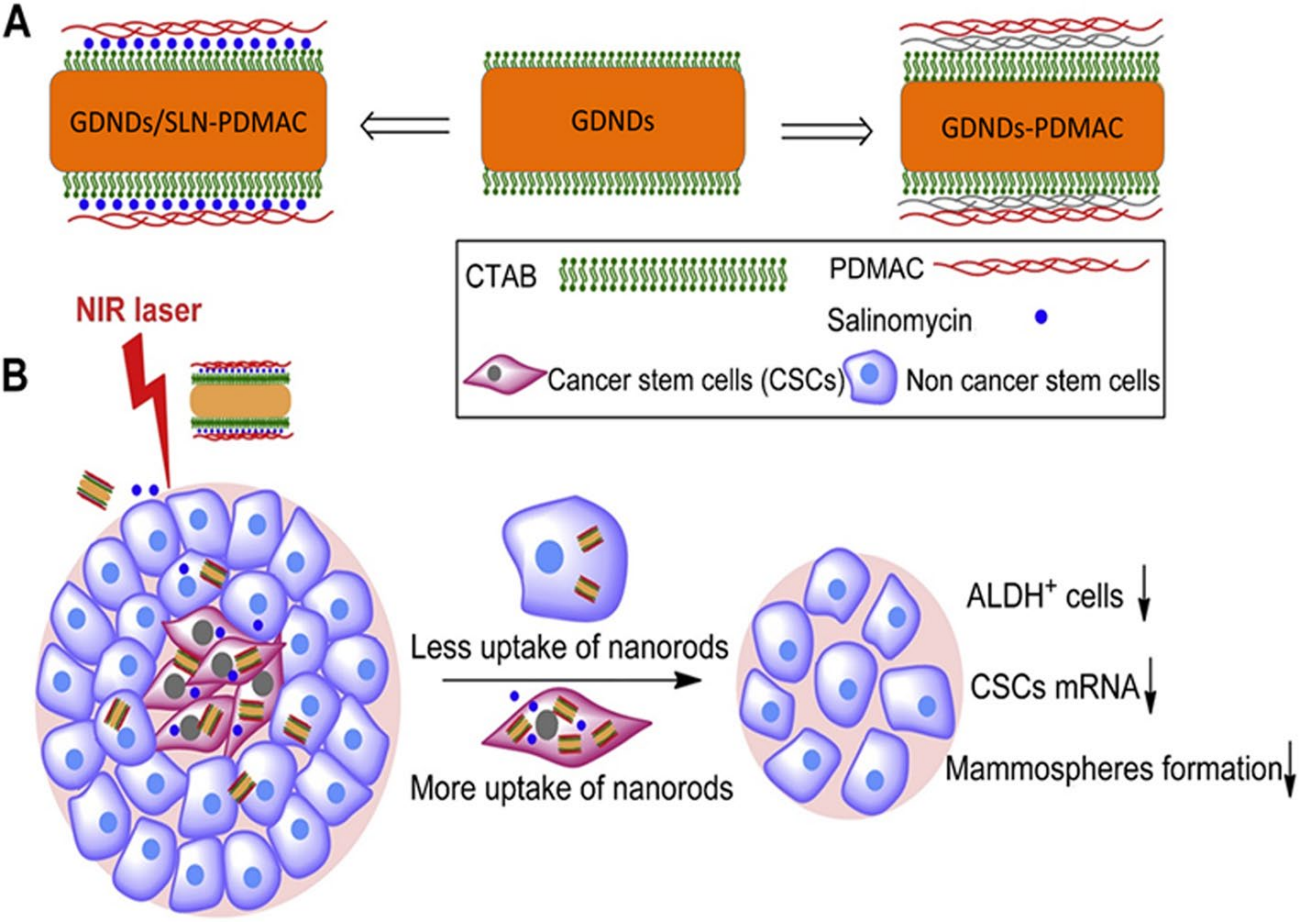
**A** Gold nanorods conjugated with polydiallyldimethylammonium chloride and loaded with salinomycin (**B**) Hyperthermia mediated by the prepared conjugation, resulting in selective elimination of cancer stem cells. Reproduced with permission from reference [109]

Cancer stem cells (CSCs), a small population of cells found inside various tumors is majorly responsible for tumor cell development and reoccurrence. Due to their profound role in hindering cancer treatment, CSCs are being recognized as potential targets for cancer therapy. However, this sub-population of cells has resistance against many cancer treatment regimens, even radiotherapy. Hence, their irradiation is a tedious task [108]. Xu and team utilize NIR-light reactive GDNDs to provide photothermal therapy that can eliminate CSCs selectively from the tumor cells. There are three chief parameters of CSCs mammospheres formation, stem cell markers gene expression, and aldehyde dehydrogenase positive (ALDHþ). The prepared GDNDs considerably suppress the mammosphere construction ability, down-regulate the expression of stem cell markers genes and constrained ALDHþ cell population in MCF-7 breast cancer cells [109]. The exterior of GDNDs was layered with polyelectrolytes, polydiallyldimethylammonium chloride (PDMAC) that is responsible for enhanced cellular uptake of nanorods by cancer cells. The same was confirmed with cellular uptake analysis, inductively coupled plasma mass spectrometry (ICP-MS) and two-photon luminescence (TPL) images verified PDMAC conjugated GDNDs were internalized at a faster rate and in a higher number by CSCs, compared to NCSCs (non-cancer stem cells) (Fig. 4), resulting in the discerning exclusion of CSCs [109]. However, the prepared GDNDs do not possess any cell cytotoxicity of their own. Hence, they were loaded with a CSC inhibitor salinomycin (SLN). This resulted in synergistic inhibition of CSCs. This synergistic activity of SLN with GDNDs was evaluated by treating MCF-7 cancer cells with SLN containing GDNDs and blank GDNDs. Both systems was accompanied by NIR radiation. Cells subjected to SLN-GDNDs + irradiation exhibit a considerable decrease in the percentage of ALDHþ cells. The total viability of cancer cells was reduced to 18%. Here, the foremost reason of cell death is the generation of heat by GDNDs via laser irradiation [109]. GDNDs facilitated hyperthermia leads to rapid active cell necrosis, bypassing the CSC’s resistance to apoptosis [110]. Hence, the CSCs aiming for thermo-chemotherapy techniques offer a more effective elimination of chemo-resistant CSCs and thus can be looked upon for overcoming resistance towards chemotherapy and cancer recurrence [109].

#### Colon cancer

Another highly dominant type of cancer with around global one million cases reported annually, colon cancer is a malignancy of the digestive system and is the second most common cause of cancer-related deaths. The rate of incidence of colon cancer is 50% higher in men than women. Diabetes, obesity, smoking, highly fat-rich diet, etc. are among the various risk factors associated with this malignancy [111].

Several phenolic compounds obtained naturally are gaining consideration in the field of cancer therapy due to their anti-oxidative and chemo-preventive properties predominantly in colon and breast cancer. One such phenol is 3,5,4’-trihydroxy-*trans-*stilbene, commonly called resveratrol [112]. When the tumor-targeting ability of resveratrol is tagged along appropriate radionuclides, this could be used to trace the site of cancer in the body. Despite these advantages, polyphenols exhibit low bioavailability, attributed to their instant and high metabolism. Therefore to overcome this issue, Kamal and the team designed AUNPs and loaded it with resveratrol radiolabeled with Technetium-99 m (TC99-RVL@AUNPs). The nanoparticles were prepared, characterized, and assessed for their tumor-targeting ability using HT29 colon cancer cells, both in vitro and in vivo [76]. The results thus obtained were then compared to corresponding outcomes obtained for TC99-RVL and TC99-AUNPs. The high drug loading (77 ± 7%) of resveratrol in the AUNPs was explained by the large surface area of the nanoparticles and the supreme intermolecular interaction between the gold particles and the drug. This is attributed to the high stability of TC99-RVL@AUNPs in excessive physiological *surroundings *in vitro. Even in ambient conditions, a shelf life of 30 days and stability after excessive dilutions (as confronted during cell line studies) endorses the easy production and storage of AUNPs and also approve their use for biological applications. Upon investigating the cytotoxic activity of TC99-AUNPs and TC99-RVL@AUNPs in rat red blood cells and HT 29 cells, both nanoparticles upto a concentration of 100 μg/ml exhibit non-toxicity on RBCs and on incubating cells with 40 μg/ml of both formulations for 24 h, > 70% cell viability was reported. A significantly higher targeting to colon cancer cells was reported by TC99-RVL@AUNPs, compared to TC99-RVL, in vivo. The study thus establishes a base for using AUNPs to deliver potent radiolabeled drugs, limited by poor bioavailability to the targeted cancer sites as theranostic agents [76].

Another greatly potent cytotoxic compound SN38 was bonded with a negative charge containing hyaluronic acid and then conjugated via electrostatic interaction to the gold nanoparticles containing a positive surface charge AUNPs @HA-SN38. Hence the prepared nanoparticles have an average particle size of 75 ± 10 nm and an overall negative surface charge. It was revealed from the release study conducted in vitro, that at pH 5.2 (acidic environment), the drug release at a faster rate compared to pH 7.4 (normal physiological condition). Also, this rate of release was elevated up to 30% using red light emitting diode (LED), compared to the study conducted in the dark [77]. To perform cytotoxicity studies, transmembrane glycoprotein MUC1 containing HT29, MUC1-deprived CHO cells and SW480 colon cancer cell lines were selected. AUNPs @HA-SN38 showed greater cytotoxicity on SW480 and HT29 cell lines than on CHO cells and the same results were confirmed by flow cytometry and confocal microscopy. After LED radiation, the incubation time for reaching IC50 was reduced to 24 h which was 48 h without LED. Also, the cell migration ability of SW480 and HT29 cells was reduced upon co-administration of LED illumination and AUNPs @HA-SN38. Increased cytotoxicity due to LED radiation was confirmed by the anti-proliferative study and this also revealed that the cytotoxicity remained for up to 192 h. Thus, AUNPs @HA-SN38 was able to overcome colon cancer metastasis effectively and has a dual anti-tumor effect. Primarily, the nanoparticle delivered the drug successfully at the site of action. The secondary effect is exhibited upon applying an external source of light, that promotes photothermal activity of the gold core, thus enhancing the cytotoxic effect of AUNPs [77].

Small-molecule inhibitors have gained immense attention in latest years. VER-155008 is a similar inhibitor that acts upon heat shock protein (HSP) 70 and 90 and down-regulates its expression, eventually promoting apoptosis [110]. HSP 70 and 90 are vital for the survival of cancer cells as it is concerned with the folding and operating of various proteins such as AKT, p53, and RAS.17. HSP over-expression at the cancer site develops resistance of tumor cells to heat leading to the ineffectiveness of PLT [113]. Hence, inhibiting these HSPs along with promoting apoptosis, reduces the heat resistance of the tumor cells [114].

Tang et al., developed a therapeutic system, comprising of HSP inhibitor-VER- 155,008 micelles (V-ms) and methoxyPEG coated GDNDs (mxPEG- GDNDs) to assess the influence of VER- 155,008 in the tumor cells on the sensitivity towards heat. Also, the therapeutic effects of V-ms along with the mxPEG- GDNDs mediated PLT were investigated. V-ms were successfully able to down-regulate HSP expression and weaken the heat-resistance developed by tumor cells [115]. The human colon cancer cell line, HCT166 was used for the in vitro analysis of the developed combination. The cells were treated with V-ms- mxPEG- GDNDs at 45 °C, a condition similar to high-temperature hyperthermia (55 °C). The apoptosis rate of V-ms- mxPEG- GDNDs treated cells was found to be 76.56%. Further in vivo studies conducted in female BALB/c nude mice showed the accumulation of V-ms- mxPEG- GDNDs inside the tumor cells, verified by fluorescent imaging and PTCI. Moreover, the size of the tumor from 2000 mm^3^ reduces considerably to less than 100 mm^3^ or even vanished when treated with the combination of V-ms- mxPEG- GDNDs along with the thermal therapy at 45 °C. Whereas when administered individually (V-ms and mxPEG- GDNDs alone) along with the thermal therapy at 45°, the tumor size reduces to around 500 mm^3^. These findings together evidenced the heat resistance attenuating property of V-ms and the synergistic activity of V-ms and mxPEG- GDNDs in solid tumor treatment [115].

Multi-drug resistance (MDR), a condition developed by cancer cells is a significant reason for the low rate of survival among advanced colorectal cancer patients. PLT is a successful method for the killing of MDR cancer cells, but cannot eliminate the tumors [116]. Jiang et al., designed a chemotherapeutic DXB-loaded GDNDs-based nanocomposite with triple layering of mesoporous silica (MS), poly-histidine (PH), and D-α-tocopherol polyethylene glycol 1000 succinate (TPLG1000) (DXB/GDNDs-MS-PH-TPLG1000) for multi-strategic targeting of MDR cells. Firstly, the GDNDs were coated with mesoporous silica (GDNDs-MS) and then loaded with DXB, this executed the photothermal-chemo-therapy combination. Further to inverse the cell resistance towards DXB, GDNDs-MS was conjugated with pH-sensitive poly-histidine. This will help in lyso/endosome escape, increasing intracellular accumulation of DXB. Furthermore, the assembly of TPLG1000 on the exterior of DXB/GDNDs-MS-PH particles will inhibit P-glycoprotein, an MDR protein, leading to intracellular retention of DXB [117].

The prepared nanocomposite in the NIR region shows favorably effective photothermal conversion, a NIR and pH activated drug release, enhanced intracellular accumulation and cytotoxic effect of DXB on SW620 human colon cancer cell line and DXB-selected ABCB1 gene overexpressing subline (SW620/Ad300). When investigated in SW620/Ad300 tumor-bearing male athymic nude mice, the nanocomposites exhibit convincing cytotoxic activity without any lethal system toxic effects, compared to other animal groups with only PLT or chemotherapy alone [117].

When the intravenous injection of DXB is injected into the patients, it produces systemic toxicity, exclusively cardiotoxicity. The DXB/GDNDs-MS-PH-TPLG1000 nanocomposite was administered intratumorally into the animals. This directed the maximum quantity of drug towards the tumor, where the drug was taken up by the tumor cells and no interaction with systemic circulation occurred [118]. Overall, the formulated nanocomposite and its potential cytotoxic efficacy may lead to the future development of similar nano-therapeutic structures for the eradication of MDR cancer cells [117].

Over past decades, core–shell fibers are gaining popularity for the delivery of chemotherapeutics, since they can avoid initial burst release [119]. Also, using pH-responsive polymers/carriers in cancer nano therapy to withstand the acidic tumor microenvironment is a well-established trick [120]. Chitosan (CSN) is one such natural polymer that is pH-sensitive and has been widely utilized for preparing matrix nano-systems that allow for rapid drug release from the matrix when reaching low pH [121]. Under similar lines, Azerbaijan and team presented the formulation of a core–shell nanofiber produced via coaxial electrospinning method by using CSN as shell and core made of poly (tetramethylene ether) glycol-based-polyurethane (PTTEG/PUE). The produced CSN- PTTEG/PUE nanofiber was then loaded with graphene oxide/ GDNDs and the chemotherapeutic drug paclitaxel (CSN- PTTEG/PUE@GG-PTX). The formulated nanofiber has dual responsiveness characteristics i.e., towards temperature and pH both. The characterization of the nanofiber system was done using a zeta sizer, Transmission electron microscopy (TEM), scanning electron microscopy (SEM), and X-ray powder diffraction (XRD) technique. The average diameter of CSN- PTTEG/PUE@GG-PXL, CSN- PTTEG/PUE@PXL, and CSN- PTTEG/PUE was 310 nm, 240 nm, and 180 nm respectively. The percentage encapsulation efficiency of PXL inside CSN- PTTEG/PUE was more than 97% [122]. The compatibility of the prepared nano-system was also inspected. Upon NIR radiation of 808 nm, the core–shell system containing graphene oxide/ GDNDs in wt.% of 10%, 20% and 50% exhibit increment in temperature from 20 °C (as initial temperature) to 51 °C, 53 °C, and 55 °C, respectively, this lead to the destruction of cancer cells as verified through in vitro analysis. The controlled release of drug from the matrix was investigated using the A549 lung cancer cell line. In in vitro cytotoxicity assay, CSN- PTTEG/PUE@GG-PXL + NIR irradiation exhibits 85% A549 cell cytotoxicity. Similarly, the in vivo studies conducted using 35 male mice verified the tumor inhibition effect of the prepared nanofiber system with NIR radiation without any alteration in the body weight of animals. However, in the free-drug group, there was a gradual decrease in the body weight of mice, indicating the adverse action of pure drugs. No change in body weight of nanofiber-treated animals signifies the targeted action of the nano-system directly on the tumor cells [122].

Targeting certain over-expressed receptors on the cancer cell surface is a well-known targeting technique in ever-evolving cancer therapy for improving cellular uptake of chemotherapeutic agents by such cells. Several studies have verified that compared with normal cells, the surface of cancer cells shows high expression of certain receptors that can be targeted to achieve a higher accumulation of drugs only in cancer cells via ligand-receptor endocytosis [123]. Transferrin receptor is one such receptor, highly expressed on the surface of certain solid tumor cells, such as lung cancer cells [124].

To target this receptor a group of scientists developed transferrin-conjugated GDNDs for the targeted delivery of DXB (TR- GDNDs/DXB). The modified GDNDs were subjected to physicochemical characterization, receptor-specificity study, hyperspectral imaging, and cancer cell uptake study. The receptor-specificity study was conducted to confirm the receptor-mediated uptake of TR- GDNDs/DXB by cancer cells. Results demonstrated the cell uptake of targeted nano-rods at 37 °C incubation and not at 4 °C. Endocytosis due to receptor-ligand interaction occurs actively at 37 °C and decreases at 4 °C [125]. This is because receptors are inactive at lower temperatures and cannot bind to their ligands [126]. Later studies conducted using HCC827 and A549 lung cancer cell lines revealed greater apoptotic, DNA damaging, and cell-killing action of TR- GDNDs/DXB rivaled to non-targeted GDNDs/DXB. The cancer cell cytotoxicity caused by non-targeted GDNDs/DXB was lower than the targeted TR- GDNDs/DXB i.e., 39% for HCC827, 36% for A549 in GDNDs/DXB and 46% for HCC827 and 48% for A549 in TR- GDNDs/DXB [125]. As shown by molecular studies TR- GDNDs/DXB exercised its cytotoxic action by caspase-9 activation [127]. Comet assay, H2AX foci, and Western blotting were done to analyze the DNA damage caused by the formulated nano-rods. A considerably greater increase in DNA laddering and H2AX foci was observed in TR- GDNDs/DXB treated cells, compared to the GDNDs/DXB treated cells group [125], representing DNA impairment and double-strand breakage of DNA [128]. Taking together, the transferrin receptor-targeted GDNDs can be used for receptor-ligand mediated targeting of cancer cells [125].

#### Lung cancer

Due to the diagnosis at an advance stage, lung cancer is a primary cause of morbidity in cancer cases. The conventional treatment of this cancer is associated with severe after-effects such as alopecia, bone marrow depression, extensive vomiting, degeneration of normal cells, etc. [129–132].

A group of scientists prepared AUNPs via the chemical reduction technique, which is a non-complex, one-step process and conjugated these AUNPs non-covalently with the chemotherapeutic drug docetaxel (DC) and folic acid (FD) was conjugated covalently to it (AUNPs@DC-FD). The chemical reduction technique allows for excellent biocompatibility, specific binding affinity and feasibility for the scale-up production of AUNPs. The physio-chemical properties of prepared AUNPs @DC-FD, DC and FD were evaluated by different analytical methods including UV–vis spectroscopy, X-ray photoelectron spectroscopy (XPS), Raman spectroscopy, X-ray diffraction (XRD), energy dispersive x-ray spectroscopy (EDS), high-resolution transmission electron microscope (HR-TEM), and field emission scanning electron microscope (FE-SEM). HR-TEM and FE-SEM were analytical tools used to determine the microstructure and surface morphology of AUNPs and AUNPs@DC-FD [133]. XRD confirmed the crystalline nature of the drug. Whereas, EDS and XPS were used to check the chemical composition and oxidation state of the prepared nanoparticles. Moreover, the cytotoxicity assay for these particles was conducted on H520 lung cancer cell line and the anti-proliferative activity of AUNPs @DC-FD, DC, FD and blank AUNPs was determined. Different concentrations of FD (5 pM, 10 pM, 25 pM, 50 pM, 100 pM, 200 pM and 300 pM), DC (20 μM, 30 μM, 40 μM and 50 μM) and AUNPs @DC-FD (10 μM, 25 μM, 50 μM, 75 μM, and 100 μM) were analyzed at different intervals of time (24, 48 and 72 h) in the selected cell line. For free DC, the IC_50_ was established at 38 μM at the 48^th^ hour and at that time interval only, the concentrations of FD and AUNPs exhibiting maximum cell death were also estimated. AUNPs at the maximum concentration of 100 μM, show 25% cell death for control and 100% viability of cells detected in the FA group at picomolar concentration. Overall, the nanoconjugates prove to be a promising and alternative carrier for treating lung and other solid cancers [133].

#### Prostate cancer

Another non-skin malignant type cancer, prostate cancer is a frequently diagnosed cancer among males. Genetic factors resulting in hormonal imbalances that result in chronic inflammation along with the and environmental factors are prominent causes of this type of carcinoma [134].

**Fig. 5:**
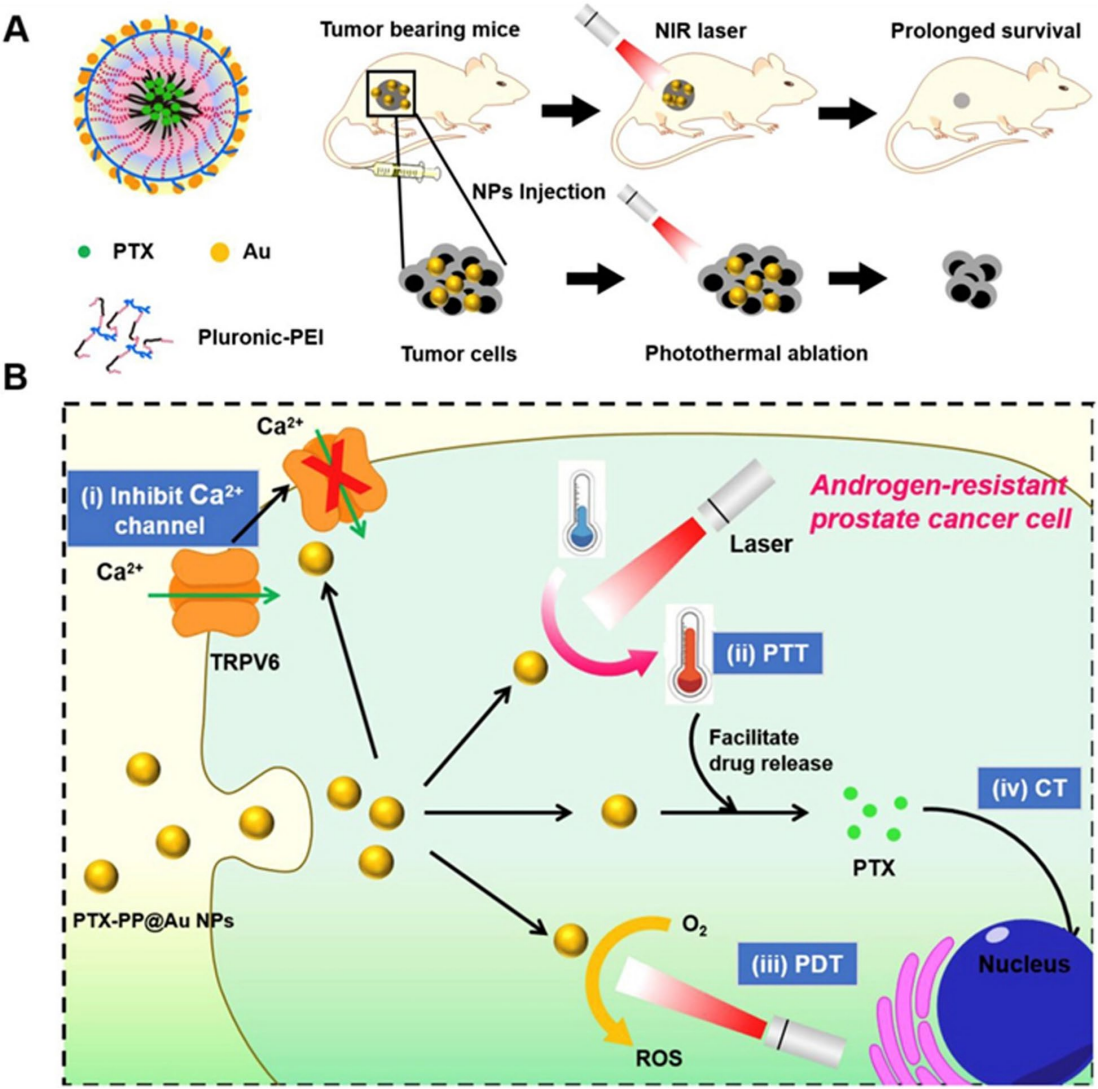
Schematic representation of (**A**) Preparation and of polyethylenimine-pluronic-paclitaxel constituted gold nanopareticles (PP-PTX@AUNPs) and its chemo-photo combinatorial therapy (**B**) Synergistic treatment by PP-PTX@AUNPs against ARPC cancer cells. Reproduced with permission from reference [82]

For advantageous curative response, prostate cancer patients are subjected to anti-androgen hormonal therapy. However, androgen-resistant prostate cancer (ANPR) emerges as an obstacle during this therapy, augmenting the metastatic process, thus leading to failure of hormonal and chemotherapy and ultimately shortening the rate of survival for patients by at least 30 months [135]. Thus, a highly efficient and non-invasive method with minimal risk is urgently required to be developed for ANPR treatment. Provided that there is no current method of treatment for ANPR, Wang et al. developed a multifunctional nanoparticle by assimilating inorganic and organic material with gold nanoparticles to exploit its properties such as carrying cargo, barricading ion channels, possessing photodynamic and photothermal effects, ROS generation. This multifunctional nanoparticle was constituted using three components: i) Paclitaxel as a chemotherapeutic agent for cancer cell arrest in early stage, ii) a cage made up of gold providing photothermal and photodynamic properties, iii) polyethylenimine-pluronic constituting a micelle to provide reduction sites for producing golden nanoparticles via “green” methodology and thus encapsulating PTX, this together forms PP-PTX@AUNPs [82]. The polymeric gold cage lead to ROS generation, temperature elevation, cation channel-TRPV6 blockage, augmenting cell cycle arrest and controlled release of encapsulated PTX. PP-PTX@AUNPs show high NIR absorbance due to photodynamic-thermal properties, which causes an elevation in temperature (Fig. 5). Also, the drug release profile showed that PTX was released from the polymeric golden cage in the 1^st^ hour but only 40% total drug was released in the 10 h, indicating immediate but controlled release activity of the formulation. ROS production in PC-3 prostate cancer cell line was analyzed using 2′,7′-Dichlorofluorescein diacetate redox probe after treating cells with PP-PTX@AUNPs for 24 h along with 5 min of NIR radiations. Current–voltage relationship of TRPV6 ions channels for demonstrating the restricted action of PP-PTX@AUNPs on these channels was established via whole-cell patch clamp tests. This ultimately causes advance tumor targeting, enhanced cytotoxicity and apoptosis of cancer cells, Thus effective therapy for ANPR with minimal invasion to other organs, as confirmed by a bio-distribution study of animals was successfully achieved via these nanoparticles [82].

Due to their localized surface plasmon resonance effect, GDNDs, possess more unique optical features than spherical AUNPs [136]. Apart from any passive discharge of loaded therapeutics that might arise from the nanoparticle surface, GDNDs with optical properties generate heat by the dispersion of absorbed light that results in the active release of pharmaceuticals. These factors have led to some success for GDNDs as medication delivery systems in experimental settings. They have been employed in a variety of biomedical fields based on these optical characteristics, such as photothermal therapy, biosensing, and gene delivery for anticancer therapy [137].

Because of the structure’s anisotropy, the GDNDS’ electrons are polarised to varying degrees in all directions. In the long axis and diameter directions, GDNDs have their own distinct electron collaborative motion. Transverse surface plasmon resonance absorption (TSPR), is the term for the resonance of electrons along the rod’s short axis within 510–530 nm range light, and longitudinal LSPR is the term for the vibration of electrons towards the longer axis of the rod with incident light that varies greatly from the visible to the near-infrared (NIR) range [138]. Because of this, the oscillation intensity of the lateral LSPR is greater and these LSPR peaks with varying aspect ratios (AR) are shifted to the NIR region rather than the visible light region by regulating synthesis, which allows dominancy of longitudinal LSPR in the optical features of GDNDs and thus the use of GDNDs in cancer imaging and therapy [139]. Different approaches utilizing GDNDs in cancer therapy are stated below (Table 2).

**Table 2:** Gold nanorods mediated imaging and therapeutic agents for treating different types of cancer

**Therapeutic agent/Bio-molecule**	**Coupling molecule**	**Type of study**	**Type of cancer**	**Name of cell line**	**Animal model**	**Outcomes**	**Ref**
Doxorubicin and photosensitizer IR820	Mesoporous organosilica, Hyaluronic acid	In vitro In vivo	Breast cancer	4T1 tumor cells	Female BALB/c nude mice	The prepared hybrid nano-system produced triple-modal-chemotherapy/Photo-dynamic/Photo-thermal combined cancer effect. The nano-system was efficiently taken up by the cancer cells due to HA & CD44 + interaction and the loaded drug get released from the system only once reached inside the tumor cells	[97]
Chlorella	Polyethylene glycol- albumin	In vitro In vivo	Breast cancer	4T1 tumor cells	BALB/c mice	The prepared Chlorella and GDNDs containing hydrogel can be effectively used to treat hypoxic tumor conditions which will lead to enhanced penetration of chemotherapeutic drugs and ultimately their cytotoxic action at tumor site	[140]
Cetuxima	-	In vitro In vivo	Breast cancer	MDA- MB-453 cells and BT-20,	BALB/c mice	The cell uptake, cytotoxicity and rate of apoptosis were significantly higher in EGFR-overexpressing BT-20 cells, both in vitro and in vivo representing EGFR-mediated targeting of prepared GDNDs	[141]
Bombesin and gastrin-releasing peptide	Polyethylene glycol	In vitro In vivo	Breast cancer	T47D breast cancer cell line, E8 human skin cancer cell line	BALB/c mice	The preparation was successfully tested against breast cancer cell lines	[142]
Salinomycin	Polydiallyldimethyl-ammonium chloride	In vitro	Breast cancer	MCF-7 breast cancer cells	-	The designed nano-system was successfully able to eradicate Cancer stem cells from tumor, in vitro	[109]
VER- 155,008	Methoxy polyethylene glycol	In vitro In vivo	Colon cancer	HCT166 Human colon cancer cell line	Female BALB/c nude mice	VER- 155,008 micelles and methoxyPEG coated gold nanorods works synergistically in inhibiting heat resistance of tumor cells and enhancing tumor cell apoptosis	[115]
Cyclic peptide GX1 and FAM172A gene	Generation-3 Poly(amidoamine) dendrimer	In vitro In vivo	Colon cancer	HCT-8 colon cancer cell line	Nude mice	PAMAM-GDNDs/GX1- FAM172A complex provides a combinatorial gene and photothermal therapy for colon cancer	[143]
Doxorubicin	Mesoporous silica, poly-histidine, and D-α-tocopherol polyethylene glycol 1000 succinate	In vitro In vivo	Colon cancer	SW620/Ad300 colon cancer cell line	Male athymic nude mice	The triple layered doxorubicin loaded gold nanorods was successfully able to overcome multi-drug resistance of cancer cells, both in vitro and in vivo	[117]
Paclitaxel	Chitosan and poly (tetramethylene ether) glycol based-polyurethane	In vitro In vivo	Lung cancer	A549 lung cancer cell line	male mice	The prepared nanofiber system led to the controlled release of paclitaxel from the polymer matrix, leading to the elimination of adverse effects of free drug in in vivo animal models	[122]
Doxorubicin	Transferrin	In vitro	Lung cancer	HCC827 and A549 lung cancer cell line	-	Transferrin-conjugated GDNDs were able to deliver the doxorubicin specially to the A549 lung cancer cells via receptor-ligand interaction	[125]
-	Anti-EGFR antibodies	In vitro	Lung cancer	A549 lung cancer cells	-	This study compared the PLT mediated by continuous and pulsed wave lasers using anti-EGFR antibodies tagged GDNDs at 850 nm	[144]
-	BAG3 gene siRNA	In vitro In vivo	Oral Squamous Cell Carcinoma	Cal-27, human oral squamous cell carcinoma cell line	Male BALB/c nude mice	The siRNA-BAG3 conjugated GDNDs nano-system with combination of PLT showed the maximum tumoroegnic effect with reduction in tumor volume to 3.8%	[145]
-	-	In vitro	Prostate Cancer	Normal prostate cell line PC-3 and prostate cancer cell lines LNCaP	-	GDNDs alter the physiology of both normal and cancer cells	[146]
N-(2-hydroxypropyl) methacrylamide (HPMA)	-	In vitro In vivo	Prostate Cancer	DU-145 prostate tumor cells	Athymic nude female mice	Radio labelled HPMA has given good results against prostate cancer when combined with GDNDs-mediated hyperthermia	[147]
Polydopamine	-	In vitro	Prostate Cancer	DU-145 prostate tumor cells	-	Improved cytotoxic effect in DU-145 cell line was observed with GDNDs conjugated to polydopamine	[148]
IL-8 silencing siRNA		In vitro	Pancreatic Cancer	MiaPaCa-2 and Panc-1 pancreatic cell lines	-	siRNA conjugated GDNDs was successfully able to silence IL-8 gene	[149]
Peptides	-	In vitro	Pancreatic Cancer	pancreatic ductal adenocarcinoma (PDA) cells	-	Higher uptake due to the conjugation of GDNDs with peptides was also observed in pancreatic cancer cells	[150]
Gemcitabine	Chitosan and mesoporous silica		Pancreatic Cancer	S2VP10 and MiaPaca2 cell line	-	Acid-sensitive chitosan-capped gemcitabine loaded-mesoporous silica-coated GDNDs were effective in enhancing the targeting specificity by using the low pH microenvironment of the tumor cell	[151]

Several experimentations are done for proposing the idea of utilizing GDNDs in prostate cancer. Musielak and associates observed toxicity on both normal (PC-3) and cancerous cell lines (LNCaP) when nanorods were used to evaluate the role of GDNDs in the response of prostate cancer and normal prostate cells to ionizing radiation on an in-vitro model. Enhanced production of ROS is one of the factors behind the effect of cytotoxicity due to nanorods. No difference was observed in the irradiation of LNCaP cells but PC-3 cells showed significantly higher irradiation when incubated in the presence of GDNDs. The absence of any changes in the proliferation of normal prostate cells has indicated the presence of some selectivity of GDNDs for cancerous cells [146].

Conjugation of Polydopamine (PM) with GDNDs was also found to be fruitful against prostate cancer when Mahmoud and associates developed polydopamine-conjugated GDNDs to assess the colloidal stability and cytotoxicity over prostate cancer cell lines. Enhanced stability of the colloid of PM conjugated with GDNDs was observed by the authors in comparison with the nonconjugated system. The cell line study over DU-145 and PC3 cell lines has established the improved cytotoxic effect of the preparation over cancerous cells. In-vitro cell migration assay has proven that GDNDs conjugated with PM is having an excellent ability to prohibit the invasion of tumor cells. Thus, PM-GDNDs conjugates could be a very good nano platform with good stability, cytotoxicity, and anti-invasion ability, to target prostate cancer [148].

#### Oral squamous cell carcinoma

Cancer of the oral cavity is a common type of malignancy. Photodynamic therapy (PCT), a renowned non-invasive cancer therapy is used widely for treating malignant diseases like oral squamous cell carcinoma [152]. Rose Bengal (RL) is a hydrophilic photosensitizing organic agent prominently used in PCT. Despite having a 76% quantum yield of singlet oxygen, RL has poor cellular uptake due to its hydrophilicity and thus is incapable of treating the solid tumor. However, being an anionic hydrophilic dye RL has an advantage in oral cancer cell targeting [153]. A group of scientists loaded RL into GDNDs (RL@ GDNDs) to combine PLT and PCT and demonstrated its anti-cancer action against oral cancer. RL@ GDNDs under NIR irradiation of 532 nm generate adequate singlet oxygen and upon increasing wavelength to 810 nm, exhibit high photothermal effect. Excellent cytotoxic effects were exhibited upon this combination of PLT and PCT, demonstrated on Cal-27 cell line. To verify the same in vivo and to resemble human oral cancer, hamster cheeks were used. Overall, in comparison to conventional oral therapies, RL@ GDNDs possess various merits. The dye-loaded nano-rods in combination with PLT and PCT showed better therapeutic effects in vitro and in vivo, compared to PLT or PCT alone. The specificity of RL towards oral cells, promotes its accumulation in cancer cells, restricting entry into normal cells. Also, when compared to free RL, RL@ GDNDs demonstrate considerably greater cellular uptake. The prepared system has outstanding cytocompatibility and upon the combination with PLT and PCT also, this method remains non-destructive and non-invasive [154].

Similarly, Darwish et al., formulated a phytochemical anticancer agent vincristine conjugated GDNDs (VC@ GDNDs) [155]. Vincristine injection exhibits great effectiveness against oral cancer upon intralesional administration [156]. However, it possesses many systemic side effects as well. So, to overcome such adverse effects, VC@ GDNDs were prepared. Silica-coated GDNDs were decorated externally with VC-loaded PEG-PLGA polymeric nanoparticles. Excellent stability of the prepared VC@ GDNDs was observed in a physiological medium of pH 7.4, in vitro. Whereas, at pH 5 which resembles TME, a sustained drug release profile of VC was obtained. For in vivo studies, oral squamous carcinoma-bearing hamster was selected as an animal model. In the control group, since no treatment was given to animals, they showed severe side effects such as overgrowth of exophytic, general debilitation, skin abscesses, hair loss and noticeable length shortening of the buccal pouch. These signs were common among control, only laser-treated and blank GDNDs treated groups. In VC aqueous solution-treated animal group, skin abscesses were lessened and tumor size was reduced moderately. In VC@ GDNDs treated group, a remarkable reduction in the size of the tumor was reported along with a low mortality rate. Furthermore, a more enhanced anti-tumor effect was observed upon treating animals with VC@ GDNDs + NIR irradiation as compared to any other individual group. Thus, this formulation successfully unloaded the potent VC selectively inside the tumor cells, hence reducing the associated side effects [155].

A negative feedback mechanism for regulating the gene expressions and metabolism, thus defining the fate of the cell is known as gene silencing [157]. siRNA or small-interfering RNA is known to interfere with specific gene expressions and may act as an efficient strategy for overcoming general issues associated with PLT, i.e. heat resistance by silencing BAG3or HSPs gene expressions. By interfering with the expression of specific genes, the small-interfering RNA (siRNA) acts as an effective vehicle in RNAinterference thereby suggesting a possible strategy to inhibit the heat shock response and render the cancer cells more susceptible to PTT by silencing the expression of heat shock proteins such as BAG3 or HSPs.

**Fig. 6:**
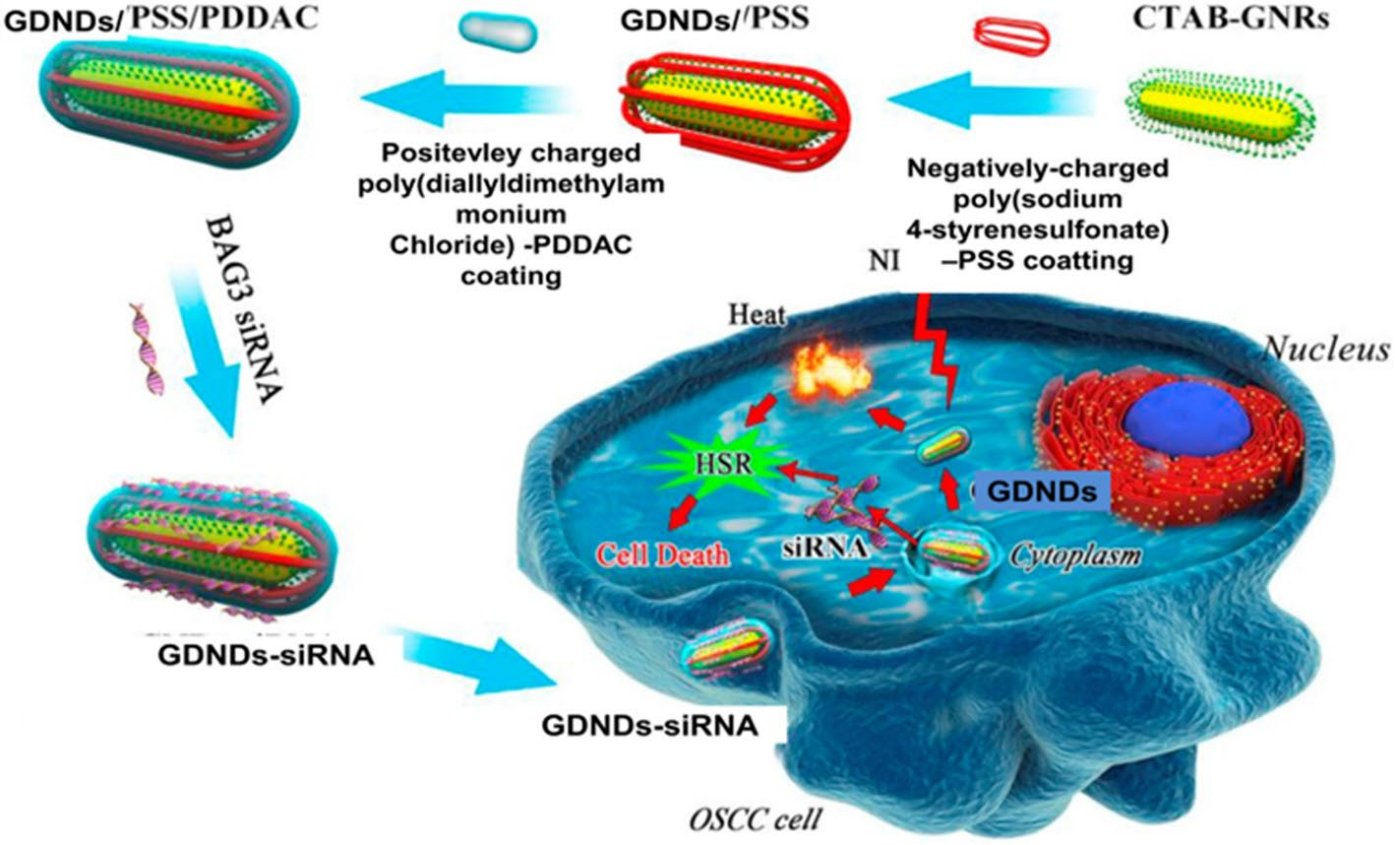
Graphical representation of the fabrication of BAG3-siRNA/GDNDs and its anti-tumor effect. Reproduced with permission from reference [145]

Given this, a team of researchers proposed the development of surface-modified GDNDs, siRNA/GDNDs system for silencing BAG3 gene and thus improving the effectiveness of PLT (Fig. 6). The developed siRNA/GDNDs upon PLT facilitation cause BAG3 silencing on both protein and mRNA levels and showed superior efficacy than the commercially available siRNA formulation, Lipofectamine 2000. The ability of siRNA/GDNDs to overcome thermoresistance and sensitize tumor cells to hyperthermia was demonstrated and compared to non-targeted GDNDs both in vitro as well as in vivo. Moderate power lasers produced by PLT were sufficient for tumor therapy in the case of siRNA/GDNDs, whereas no effect was seen in the non-targeted group by same-intensity laser waves [145]. This is advantageous as lasers of low power are more tolerant to patients, reduce the associated side effects of PLT, and lessen the chances of overheating-related dysfunctioning inside the human body [158]. Also in the absence of laser therapy, siRNA/GDNDs do not exhibit any cytotoxicity, both in vitro* and *in vivo, signifying their biocompatibility. No effect on normal cells was reported due to low BAG3 expression in these cells indicating the targeting ability of the prepared nano-system [145].

In vivo study conducted using Cal-27, human oral squamous cell carcinoma containing male BALB/c nude mice demonstrated the tumoricidal efficacy of siRNA/GDNDs. The animals were divided into 5 groups and intratumorally treated with different systems. Group 1 was saline as the control group, Group 2 was administered with BAG3-siRNA and given PLT, no inhibition of tumor growth versus saline was observed in this group, indicating no cytotoxic effect of laser or siRNA or laser alone. Group 3 was treated with BAG3-siRNA/GDNDs, without PLT.T the tumor growth in this group slightly reduces to 79.5% compared to the control, signifying the delivery of siRNA by GDNDs and tumor suppression effect after BAG3 gene silencing. GDNDs + PLT group was numbered 4. The tumor growth after 18 days was highly suppressed in the animals, reducing tumor volume to 39.8%. However, regrowth occurred after 10 days of therapy. Group 5 animals were treated with BAG3-siRNA/GDNDs + PLT for 18 days and this group showed the maximum inhibition of tumor growth with tumor volume reduced by up to 3.8% with no resume of growth after the treatment ended. This study indicated the suitable and controllable gene silencing efficiency of the GDNDs [145].

Camouflaging nanoparticles with natural cell membranes is a recently growing strategy in various malignancies including cancer [159]. These camouflaged nanoparticles show prolonged circulation in the blood and get selectively accumulated inside the tumor cells as they inherent characteristics of the cell membrane [160]. Nanoparticles coated with membranes of cancer cells exclusively, because of their unique membrane structure and composition can overcome nonspecific binding, defeat immune clearance and get benefits such as homologous binding ability and immune escape [161]. Taking advantage of this concept, Sun and co-workers fabricated GDNDs with KB cell line obtained oral squamous cancer cell membrane (GDNDs/KB). The fabricated nano-rods upon X-ray irradiation possess radiosensitizing effect and in the second NIR window exhibit outstanding photothermal transfer ability. Upon X-ray and NIR irradiation, GDNDs brought an increase in temperature, and ROS generation, which ultimately damages the DNA helix of cancer cells, inducing apoptosis. The coating of GDNDs with cancer membrane stabilizes the nano-system in the physiological environment and benefits in vitro-specific cancer cell targeting. Relatively longer blood circulation, cancer cell targeting, and tumorigenicity of GDNDs/KB were demonstrated in male nude mice as well [162].

The tumor-inhibiting study conducted for 28 days demonstrated that no growth inhibition was exhibited by NIR irradiation group alone, similar to the control group with tumor volume reaching up to 2275 mm^3^ and 2368 mm^3^ in NIR and control groups respectively. In GDNDs/KB + NIR irradiation-treated animals, the tumor growth was much slower, measuring 731 mm^3^. This is attributed to the higher tumor cell accumulation of GDNDs/KB. Additionally, with GDNDs/KB + NIR + X-ray irradiation, there was the complete eradication of tumor cells was observed in 4 animals out of 5, signifying the most potent inhibition acton of this therapy regimen. Thus the prepared GDNDs/KB revealed robust cancer cell-killing activity [162].

#### Pancreatic cancer

With a survival rate of almost 5%, pancreatic cancer is an extremely fatal disease, resulting in more than 30,000 deaths in United States annually. Inadequate rate of prognosis and high rate of mortality of pancreatic cancer endorse both intrinsic and extrinsic drug resistance [163]**.** RNAi is used to prevent the overexpression of Interleukin-8/IL-8 by gene silencing in the case of pancreatic cancer, the efficacy of which is found to increase when GDNDs were used for the delivery of RNAi by Panwar et al. They prepared nanorods with positive zeta potential which can be easily attached to the negatively charged siRNA. Good gene knockdown capability of IL- mRNA was established by pancreatic cell line studies over MiaPaCa-2 and Panc-1 cell lines. The in-vitro study has suggested potentially good uptake of siRNA-loaded nanorod [149].

Higher uptake due to the conjugation of GDNDs with peptides was also observed in pancreatic cancer cells in the study of the effect of multifunctional GDNDs for selective plasmonic PLT, done by Patino et al. The use of GDNDs drastically improved the stability of the colloid which can help to avoid the related toxicity of nanorods. The study has suggested that GDNDs are having a very good ability to localize the hyperthermia generated by plasmonic PLT, which can cause heavy destruction to the cancerous cells [150].

Furthermore, Zeiderman and associates have prepared acid-sensitive chitosan-capped gemcitabine loaded-mesoporous silica-coated GDNDs to enhance the targeting specificity by using the low pH microenvironment of the tumor cell. An In-vitro study of gemcitabine-loaded nanorod preparation over S2VP10 and MiaPaca2 cells has shown higher cytotoxicity in comparison with the gemcitabine alone. S2VP10 and Miapaca2 cells have shown 97%, 96.5% for gemcitabine-loaded nanorod preparation and 60%, 76% for gemcitabine alone respectively. A higher accumulation of prepared formulation was observed by multispectral optoacoustic tomography (MSOT) in the in-vivo S2VP10 model. Tumor targeting and drug conveyance of the nanorods were made easy due to the pH responsiveness of the peptide and polymer coating over the particles [151].

### Immunotherapy

In recent years, immune checkpoint blockade therapy has emerged as a promising approach in cancer treatment. Immune checkpoint inhibitors (ICI), disrupt the immune checkpoint signals, augmenting one’s immune system to fight against tumors [164]. Tumor cells suppress the expression of co-stimulatory surface antigens and molecules, which lowers T cell activation and its recognition. Additionally, cancer cells release immunosuppressive cytokines including TGF and IL-10, which disrupt the environment for the maturation of dendritic cells (DCs). Additionally, they possess the capacity to produce substances like FasL and TRAIL that cause death of T cells [165]. Numerous advances have been witnessed which demonstrated the emergence of chimeric antigen receptor (CAR) T cell and ICI-based therapies. Dozens of immunotherapeutic against cancer have been approved which include tisagenlecleucel [166], durvalumab [167], ciloleucel and axicabtagene [168].

Xiao et al., worked on colorectal cancer and combined the promising features of photothermal therapy and immunotherapy. Herein, biomimetic polydopamine (PDA) decorated gold nanostar nanoparticles (GNS) were manufactured and finally functionalized with anti-programmed ligand-1 (PD-L1). PDA-GNS were opted for due to the large surface area, remarkable photothermal conversion ability, near-infrared absorption ability and photothermal stability. The nanoparticles elevated the maturation of dendritic cells, reduced the expression of myeloid-derived suppressive cells and T cells, and enhance CD8 + T cell infiltration to modify the environment of murine cancer models. Additionally, the approach demonstrated improved therapeutic efficacy, considerably limiting tumor growth and increasing overall survival. The photothermal response was observed using UV–vis spectroscopy method, which showed a slight red shift in peak from 848 to 865 nm, which could be due to a change of refractive index due to the coating of the nanoparticle. As compared to PDA-GNS, PDA/GNS@aPD-L1 showed profound photothermal tumor-killing effects in vitro due to stronger binding capability. Moreover, coating with membrane improved the cell internalization ability of the therapeutic system. In vivo*,* the response demonstrated photothermal ablation and PD-1-PD-L1 blockade due to synergistic performance by PDA/GNS@aPD-L1. One of the most significant antigen-presenting cells, dendritic cells are the initial phase of adaptive immune responses, which are essential for anti-tumor immunity. They also have a distinctive capacity to trigger naive T cells. In the inguinal lymph nodes, the maturity of DC (CD11c + CD86 + CD80 +) was assessed and discovered that the proportion of mature DCs was slightly elevated (31% for PDA/GNS@aPD-L1) as compared to PBS treated groups. The PDA/GNS@aPD-L1 NP + NIR group had the highest ratio CD4 + T to CD8 + cells in the lymph nodes and spleen, demonstrating increased differentiation of naive T cells into CD8 + T cells and considerable stimulation of the systemic immune system. Overall, the therapy of the dual approach excellently demonstrated inhibitory effects both in vitro and in vivo [169].

Another similar kind of study was published by Wang et al. PD-L1 antibody-modified gold nanocages loaded with galunisertib (TGFβ inhibitor) and conjugated with macrophage membrane showed inhibition of distant tumor growth and eliminated primary tumor mass under the influence of abscopal effect. The action was more elevated under the effect of PTT. CT-26 cells after treatment with the developed system suitably downregulated the heat shock proteins (HSP90 and HSP70), which are highly responsible for thermos-resistance by cancer cells [170].

The tumor microenvironment demonstrates a vital constituent called tumor-associated macrophages (TAM) which polarizes under specific stimuli. M1 and M2 phenotypes are the two extremes of the polarization of macrophages. The majority of TAMs have a characteristic M2 phenotype, which could greatly accelerate the development of tumors due to increased production of immunosuppressive molecules such as vascular endothelial growth factor (VEGF), transforming growth factor-beta (TGF-β) and interleukin-10 (IL-10). Zhang et al., developed poly (ethylene glycol) conjugated AUNP that inhibited TAM M2 polarization through autophagy intervention. As PEG-AuNPs cause membrane permeabilization and lysosome alkalization, they have the potential to limit autophagic flux in TAMs. Additionally, once autophagy was activated, TAMs polarized towards the M2 phenotype, whereas reduction of autophagic flux might limit this polarization. Hence, immunomodulation by nanoparticle plays an important role in developing potent therapeutic results.

### Radiotherapy

Radiotherapy in solid tumors is greatly limited by the development of radioresistance. Over time, AUNPs s have emerged as promising radiosensitizers for tumor-bearing patients [171]. So to investigate the mechanism and radiosensitizing ability of siRNA-loaded AUNPs on lung tumors, Zhuang et al. designed SP1-siRNA tagged AUNPs (AUNPs -SP1) [81]. SP1 or Specificity Protein 1 is an overly expressed protein present on the liquid-based cells of the bronchial brushings [172]. SP1 -siRNA was attached non-covalently to AUNPs and the capability of AUNPs to adsorb siRNA was estimated via gel electrophoresis. Confocal microscopy confirms the uptake of AUNPs -SP1by A549 cells and to validate the silencing activity of siRNA, western blotting assay and RT-qPCR was conducted. Plate colony formation analysis validates radiosensitization and CCK-8 analysis estimated the viability of cancer cells after incubation for 24 h with AUNPs -SP1 [81]. A study revealed that overexpression of granzyme B (GRYE-B) may make cell lung cancer cells more vulnerable to natural killer cell-facilitated killing [173]. Therefore, to confirm the breaking of double strands of DNA in the absence and presence of GRYE-B and AUNPs -SP1, by immunofluorescence study was conducted. The mechanism of downregulating SP-1 was foreseen in bioinformatics assays. Radiation sensitivity of GRYE-B and AUNPs -SP1 was verified in vivo by establishing tumorigenesis in balb/c mice, subcutaneously. SP1-siRNA was successfully absorbed on AUNPs -SP1 surface and efficiently internalized in A549 cells, thus down-regulation expression of SP1 protein. AUNPs -SP1 encouraged cancer cells to enter G2/M phase of the cell cycle, breaking the double bonds of DNA, ultimately achieving radiosensitization. Moreover, AUNPs -SP1 significantly inhibited the tumor growth in vivo, by hindering SP1 expression and up-regulating GRYE-B [81].

Localization of N-(2-hydroxypropyl) methacrylamide (HPMA) was observed to be increased when GDNDs were used for activating controlled heat generation to create localized hyperthermia. Radio-labeled HPMA has given good results against prostate cancer when combined with GDNDs-mediated hyperthermia synthesized by seed-mediated growth method. Localized hyperthermia created by the PLT can be initiated and controlled by GDNDs. The use of nanorod-mediated hyperthermia to enhance the effect of radiotherapy was observed in the efficacy study. They have observed an extra suppression of tumors which may be due to the overall increase in temperature around the GDNDs [147].

### Angiogenesis arrest

Angiogenesis denotes to development of blood vessels from pre-existing ones which is mediated by the interaction between pro- and anti-angiogenic agents. For cancer development, nutrients and oxygen are highly necessitated by the uncontrolled growth of cancer cells. As a result, cancer cells release a variety of growth factors to promote neo-angiogenesis, which subsequently helps supply the body with much-needed nutrients. Several FDA-approved anti-angiogenic agents have demonstrated the ability to reduce tumor-induced neo-angiogenesis, nevertheless, their effectiveness is hampered by significant adverse effects on other organs’ normal vascular architecture and the emergence of drug resistance [174, 175].

Nanoparticles hold great potential in delivering cargo selectively to the cancer cells by enhanced permeation and retention (EPR) at the cancer site. The key players of the tumor microenvironment include endothelial cells (EC), cancer associated fibroblast (CAFs) and cancer cells (CC). The crosstalk among such players plays a significant role in cancer metastasis and resistance. Inhibition of such crosstalk was demonstrated by AUNPs in vitro. Reduced effects on ECs tube development and migration are seen in the conditioned media (CM) of cells that have been pretreated with AuNPs or cells that have been cocultured with them. AuNPs remove up to 45% of VEGF165 from CM and 95% of VEGF165 from VEGF single-protein solution, which results in reduced VEGF-Receptor 2 (VEGFR2) activation compared to control CM. These findings showed that AuNPs impede VEGF-VEGFR2 signaling to endothelial cells from TME cells to prevent angiogenesis [176]. In another study, quercetin-loaded AUNP inhibited angiogenesis by targeting the EGFR/VEGFR-2 signalling pathway as compared to free quercetin in DMBA-induced mammary carcinoma rat model.

## Theranostic applications of gold nanoparticles

The term theranostic combines both revolutionizing the clinical field- therapy and diagnosis. These two target-one arrows-based approaches is an effective way to tackle the most deleterious disease as conventional technique of diagnosis and imaging methods make it impossible to achieve desired results. The imaging technique can be improved by the use of nanoparticles due to their unique magnetic and optical properties. Also, a wide range of therapeutic as well as diagnostic abilities exhibits multifunctional benefits in cancer therapy.

Recently, as a smart way of treatment, Taghavi et al., brought up MUC1 aptamer functionalized, mesenchymal stem cell membrane (MSCM) coated, DXB loaded gold nanoparticles which illicitly demonstrated profound targeting behavior against MUC1 positive cell lines (4T1) showing mortality at 0.468 µg/ml and 0.23 µg/ml taken an equivalent dose of DXB, however, such cytotoxicity wasn’t observed in MUC1 positive cell lines. Even after 24 h of IV injection, the targeted therapy showed intense tumor accumulation. Thanks to the hollow gold nano platform that enabled CT scan imaging in 4T1-laden cancer cells in vivo. The designed paradigm outbursted the theranostic scope of gold nanoparticles [177].

Strategizing the drug delivery system to a level that shows no toxic reactions, rapid uptake and improved cellular inhibition assists in cell death. Nanoparticles should have a size greater than 10 nm and should be about 100 nm to avoid renal filtration and specific liver uptake, respectively. AUNP can easily be constructed in the desired range, however, colloidal stability caused aggregation of nanopreparation, resulting in long-term instability. It is therefore essential to precisely control the functional group and remove the residual contaminants arising during the synthesis of the particles. Yilmaz et al., loaded DXB into a modified form of AUNP which was coated with polymethacrylic acid (PMAA) having a cysteine linkage forming PMAA-AUNP-Cys-DXB conjugate. The cell images obtained from confocal microscopy showed increased nucleus localization as compared to the free dox [178]. Thus, it could be established that discovering the wide range of theranostics using gold nanoparticles help detect cancer cells at an early stage.

## Conclusion

This review brings the latest research conducted using gold nanoparticles (AUNPs), specifically gold nanorods (GDNDs) for combating different types of cancer. The general properties, optical characteristics, ease of synthesis, size and shape modification feature of this metallic system make it a promising candidate for cancer diagnosis, imaging and therapy. However, like other nano-systems, AUNPs/GDNDs also possess certain challenges and disadvantages that are needed to be addressed. Further research is necessary before adopting multi-modal and multifunctional AUNPs/GDNDs for clinical use. By far, the observations made are based on the in vitro and animal models data, leaving behind a question mark for the action of this system upon human exposure, at both laboratory levels and commercial levels. Validating the in vivo fate of AUNPs/GDNDs, will help in predicting the pharmacodynamics of the system. Furthermore, nanotoxicity assessment is also required to be evaluated to confirm the suppression of one of the biggest disadvantages of chemotherapy, i.e. associated side effects. The particle size is one of the factors responsible for showing toxicity of AUNPs, specifically the smaller ones. This is due to their ability to cross the cell membrane and reach the nucleus more rapidly. Findings also suggested that a size smaller than 5 nm can rapidly be removed through the urinary system. Apart from size, another major key factor is the shape of nanoparticles. GDND had a more toxic effect on the zebrafish model as compared to the spherical ones, however, such an effect can be tackled by coating the GDNDs with materials such as polyethylene glycol or phosphatidylcholine. Biocompatibility is another issue which is needed to be tackled as cytotoxicity depends on numerous parameters such as cell type, tissue distribution, type of cell, etc. The production cost of AUNPs limits its marketing growth. Xia and the team suggested that the cost of photothermal therapy upon using PEGylated AUNP is very high [179]. Furthermore, AUNPs are still in the preclinical stage, with only a few cases of laboratory investigations being translated into clinical trials. More research is needed to recreate the actual scenarios of tumor occurrence and understand the mechanisms in concern with the medical applications. Overall, additional theoretical discoveries and exploration of the capabilities of gold nanoparticles in TME applications are required to promote the process of nanotechnology implemented in greater medical practice. Despite the chemical inertness, gold is a noble metal with inherent chemical toxicity up to an extent. Although the addition of functional moieties such as stabilizing and biocompatible materials appears to reduce the toxicity of AuNP, it is important to note that a variety of surface changes may also generate undesired side effects. More study is needed to assess the trade-off between treatment or diagnosis benefit. It is also critical to investigate whether and how the functional chemicals alter biodistribution and the resulting adverse effects. Similarly, surface modification technology must be developed if we are to prevent the epidemic of immunogenicity in vivo. Another potential influence on biosafety is the biodistribution of AUNP due to their accumulation in the liver, spleen, and other organs, causing harmful consequences in these organs. More research is needed to fully comprehend the biodistribution characteristics of AuNPs. More targeted nanoparticles are needed to be developed to escort them directly into the cells and spare the normal cells. Cell targeting reduces the dose of the drug, overcomes drug resistance, and avoids rapid removal. Hence, more clinical studies using targeting nanosystems can help reach the clinical markets. A systemic risk–benefit evaluation of this theranostic system is essentially needed before proposing AUNPs/GDNDs for clinical application.

## Data Availability

Not applicable.
